# Conditional Ablation of Glucocorticoid and Mineralocorticoid Receptors from Cochlear Supporting Cells Reveals Their Differential Roles for Hearing Sensitivity and Dynamics of Recovery from Noise-Induced Hearing Loss

**DOI:** 10.3390/ijms24043320

**Published:** 2023-02-07

**Authors:** Charles C. Barnes, Kathleen T. Yee, Douglas E. Vetter

**Affiliations:** 1 Graduate Program in Neuroscience, University of Mississippi Medical Center, Jackson, MS 39216, USA; 2 Department of Otolaryngology–Head and Neck Surgery, University of Mississippi Medical Center, Jackson, MS 39216, USA

**Keywords:** mineralocorticoid receptor, glucocorticoid receptor, Nr3c1, Nr3c2, cochlea, conditional gene ablation, noise-induced hearing loss, cochlear support cells

## Abstract

Endogenous glucocorticoids (GC) are known to modulate basic elements of cochlear physiology. These include both noise-induced injury and circadian rhythms. While GC signaling in the cochlea can directly influence auditory transduction via actions on hair cells and spiral ganglion neurons, evidence also indicates that GC signaling exerts effects via tissue homeostatic processes that can include effects on cochlear immunomodulation. GCs act at both the glucocorticoid receptor (GR) and the mineralocorticoid receptor (MR). Most cell types in the cochlea express both receptors sensitive to GCs. The GR is associated with acquired sensorineural hearing loss (SNHL) through its effects on both gene expression and immunomodulatory programs. The MR has been associated with age-related hearing loss through dysfunction of ionic homeostatic balance. Cochlear supporting cells maintain local homeostatic requirements, are sensitive to perturbation, and participate in inflammatory signaling. Here, we have used conditional gene manipulation techniques to target Nr3c1 (GR) or Nr3c2 (MR) for tamoxifen-induced gene ablation in Sox9-expressing cochlear supporting cells of adult mice to investigate whether either of the receptors sensitive to GCs plays a role in protecting against (or exacerbating) noise-induced cochlear damage. We have selected mild intensity noise exposure to examine the role of these receptors related to more commonly experienced noise levels. Our results reveal distinct roles of these GC receptors for both basal auditory thresholds prior to noise exposure and during recovery from mild noise exposure. Prior to noise exposure, auditory brainstem responses (ABRs) were measured in mice carrying the floxed allele of interest and the Cre recombinase transgene, but not receiving tamoxifen injections (defined as control (no tamoxifen treatment), versus conditional knockout (cKO) mice, defined as mice having received tamoxifen injections. Results revealed hypersensitive thresholds to mid- to low-frequencies after tamoxifen-induced GR ablation from Sox9-expressing cochlear supporting cells compared to control (no tamoxifen) mice. GR ablation from Sox9-expressing cochlear supporting cells resulted in a permanent threshold shift in mid-basal cochlear frequency regions after mild noise exposure that produced only a temporary threshold shift in both control (no tamoxifen) f/fGR:Sox9iCre^+^ and heterozygous f/+GR:Sox9iCre^+^ tamoxifen-treated mice. A similar comparison of basal ABRs measured in control (no tamoxifen) and tamoxifen-treated, floxed MR mice prior to noise exposure indicated no difference in baseline thresholds. After mild noise exposure, MR ablation was initially associated with a complete threshold recovery at 22.6 kHz by 3 days post-noise. Threshold continued to shift to higher sensitivity over time such that by 30 days post-noise exposure the 22.6 kHz ABR threshold was 10 dB more sensitive than baseline. Further, MR ablation produced a temporary reduction in peak 1 neural amplitude one day post-noise. While supporting cell GR ablation trended towards reducing numbers of ribbon synapses, MR ablation reduced ribbon synapse counts but did not exacerbate noise-induced damage including synapse loss at the experimental endpoint. GR ablation from the targeted supporting cells increased the basal resting number of Iba1-positive (innate) immune cells (no noise exposure) and decreased the number of Iba1-positive cells seven days following noise exposure. MR ablation did not alter innate immune cell numbers at seven days post-noise exposure. Taken together, these findings support differential roles of cochlear supporting cell MR and GR expression at basal, resting conditions and especially during recovery from noise exposure.

## 1. Introduction

Noise exposure is a major contributor to acquired sensorineural hearing loss and is second only to age-related hearing loss in the number of individuals affected. Currently, there are no FDA-approved drugs that reduce noise-induced hearing loss (NIHL). A significant unmet medical need worldwide is the production of advanced therapeutics designed to reduce NIHL, which is a permanent loss of auditory frequency representation because lost cochlear hair cells are not replaced in the mammalian cochlea. Currently, in human patients, glucocorticoids (GCs) are administered to reduce immune-related hearing losses such as those generated from cochlear implantation, sudden sensorineural hearing loss (SNHL), and acutely after noise exposure (reviewed by [1]). The justification for using GCs in an attempt to ameliorate hearing loss produced via such disparate challenges stems from the idea that inflammation plays an integral role in injury and recovery cycles. To date, however, therapeutic outcomes following GC treatments unfortunately only result in mixed success when treating forms of hearing loss [2]. A better understanding of the dynamics and processes served by GCs in the cochlea, especially with respect to any role leading to maintenance of normal auditory function in the face of acute challenges, could suggest novel interventional strategies.

In rodents, the major bioactive corticosteroid is corticosterone (Cort), which is typically released from the adrenals in a circadian rhythm and in response to stress. There are two ligand-activated nuclear receptors sensitive to corticosteroids—the glucocorticoid and mineralocorticoid receptors (GRs and MRs, respectively). While the MR has high affinity for the major mineralocorticoid aldosterone, it has an equally high affinity for Cort. Both the MR and GR are expressed in many cochlear cell types [3]. MR can exert rapid effects through various signaling cascades and slow effects through transcriptional regulation (reviewed by [4,5]. The GR is broadly expressed throughout the body, but despite its name has roughly ten times lower affinity for Cort than does the MR.

In cells expressing both MR and GR, the MR is the first Cort-responsive system activated, with GR signaling occurring only after the MR pool has been saturated. For example, at times of peak circulating Cort levels such as that caused by stress or during certain cycles of the circadian program, Cort levels will surpass the ability of the MR pool and spill over to the GR pool. Thus, when GR is activated in cells that also express MR, it will indicate (code for) increased Cort levels. Thus, functionally, the MR:GR ratio has been considered crucial for normal adaptation to constantly changing environmental stressors at the organism, tissue, and even cellular level for those cells expressing both receptors (reviewed by [6]). Within the auditory system, stimulated GC signaling in the cochlea activates GR to initiate anti-inflammation programs through NF-κB inhibition [7,8,9], while stimulated MR activity may improve auditory function through stabilization of ionic and water balance [10,11]. Unique combinations of MR and GR expression and intracellular signaling components can produce cell-type-specific responses. For example, steroid-responsive gene expression is differentially regulated across cells within the stria vascularis, organ of Corti, and spiral ganglion neurons (SGN) [12]. Indeed, the systemic stress axis and noise exposure modify spiral ganglion neural adaptation potentially through the sensitivity of MR and GR expressed in SGN [13,14]. Importantly, synthetic GC signaling produces effects rooted in broader cochlear homeostatic functions implicating multicellular, tissue-based adaptation.

The innate immune response is a well-known process involved in recruiting multiple cell types to a site of injury and contributes to inflammation for tissue maintenance and ultimate recovery after injury. The cochlea harbors resident immune cells and is the target of immune response which alters recovery of the damaged cochlea [15,16,17,18,19]. Noise exposure generates glutamatergic excitotoxicity and metabolic oxidative stress as primary generators of cellular damage driving cochlear inflammation and immune response. Cort levels naturally cycle with the circadian rhythm and are positively correlated with NIHL susceptibility and proinflammatory signaling [20]. A circadian influence on NIHL in mice appeared distinct from noise-driven excitotoxicity, suggesting multicellular patterning of GC-related cochlear response to noise exposure. Inflammatory response, the dominant response of the cochlea to noise [21], is thought to be contributed in part by supporting cells [22]. Given that GC-based effects on cochlear homeostasis are likely to be important in shaping responses to stressors such as noise while also being influenced by the circadian state, further investigation of potential causal links between GC signaling and homeostatic programs in the cochlea is warranted.

The complex multicellular organization of the cochlea underlies its sensory functionality. The constant functional responsiveness to sound coupled with the lack of regenerative abilities of the sensory cells highlight the requirement for surveillance of potential homeostatic challenges to the cochlea that invoke cellular programs responsible for restoring homeostasis. Thus, the specialized sensory function of the cochlea is made possible only by the coordinated activities of the non-sensory “supporting cell” structures of the cochlea. These cells maintain the basal physiology of the cochlea to allow sensory transduction to occur. The cochlear “supporting cells” are an organization of heterogeneous non-sensory cell types that line the scala media (we exclude from consideration here those cells of the spiral ligament) and are sensitive to the local cochlear micro-environment (reviewed by [23,24,25]). Most supporting cell types are stress responsive and contribute to cochlear inflammation through the expression of pro-inflammatory mediators [22,26,27]. Indeed, supporting cells are known contributors to direct and indirect GC-signaling effects within the cochlea that include inflammation and immune response resolution [28]. Co-expression of GR and MR suggests a potential for overlapping and complementary effects of GC signaling in most cochlear cell types [3]. Since supporting cells near the organ of Corti can assume macrophage-like functions [29], MR activity in supporting cells may be proinflammatory as other studies indicate for myeloid and microglia cells [30,31]. However, data on MR activity resolving inflammation seem to be related to cerebral ischemia [30] and in the cochlea, to conditions related to autoimmune disorders [32] and processes involving ionic imbalance such as hydrops [33,34].

Systemically, release of corticotropin releasing factor (CRF) by the paraventricular nucleus initiates a signaling system across the hypothalamic–pituitary–adrenal (HPA) axis to induce adrenal release of glucocorticoids such as cortisol (in humans) and corticosterone (in rodents) and the mineralocorticoid, aldosterone. These products of CRF signaling bind to the GR and MR. Both GR and MR are widely expressed in many tissues/cells of the body. Our early work on CRF-like urocortin signaling in the cochlea revealed heavy expression of CRF receptors in the non-sensory supporting cells lining the scala media [35], suggesting that intercellular signaling via CRF-like peptides may represent a local HPA-like signaling system. Because GR and MR play major roles in general physiology throughout the body and may have complementary or overlapping roles in the cochlea, an experimental approach must be used to separate the roles for systemic GR and MR signaling from the roles of GR and MR in the cochlea to allow specific conclusions to be drawn about cochlear MR and GR function in the maintenance of normal cochlear functional and structural states at rest and following homeostatic challenges. Given that systemic HPA signaling is initiated via CRF signaling for ultimate release of GCs, and that in the cochlea the vast majority of cells susceptible to CRF-like signaling are the supporting cells lining the scala media, we adopted a cell-specific, inducible gene knockout strategy designed to parse MR and GR function from these supporting cells likely participating in CRF signaling. It has been shown that Sox9 is expressed in supporting cells of the inner ear but not hair cells or ganglion cells [36,37,38,39,40,41] and thus the Sox9 gene may represent a useful driver for Cre recombinase expression by which spatially and temporally targeted gene manipulation can be produced specifically in cochlear-supporting cells.

The specific goals of the current study were to assess the influence of mineralocorticoid and glucocorticoid receptors expressed in cochlear non-sensory-supporting cells on basal threshold sensitivity, the dynamics of threshold recovery from noise exposure while leaving systemic GC signaling intact, and the immune cell response to noise exposure. Given the correlation between cochlear supporting cells and various aspects of homeostatic response to stressors, we sought to target manipulation of MR and GR expression in those cells and define the role of the corticosterone-related stress response emanating from these cells by way of MR and GR activation. Specifically, we investigated the roles of supporting cell MR and GR on the susceptibility to NIHL and auditory functional recovery after mild noise exposure of naïve mice. We used targeted cell-specific, inducible genetic knockout mouse models to eliminate MR or GR expression from Sox9-expressing cochlear-supporting cells. We assessed cochlear functional and structural integrity following mild noise exposure by examining auditory brainstem responses (ABRs), 2f1–f2 distortion product otoacoustic emissions (DP’s), the degree of synaptopathy following noise by examining the pairing of pre- and post-synaptic afferent elements associated with IHCs, and finally the cellular aspects of immune response/inflammation by examining the presence and phenotype of Iba1-positive immune cells at 7 days post-noise exposure. We found that supporting cell GR ablation induced noise susceptibility distinct from ribbon synapse vulnerability. Supporting cell MR ablation was dispensable for stable recovery from noise exposure but instead was associated with an acute but transient ABR P1 reduction. This study is the first to divorce the roles of MR and GR, the dual receptors for GC signaling, in the cochlea and suggests distinct functional roles for expression of MR and GR in cochlear-supporting cells for basal auditory brainstem thresholds and the ability for recovery from noise-induced hearing loss.

## 2. Results

### 2.1. Sox9iCre Drives Gene Recombination in Cochlear-Supporting Cells

**Figure 1:**
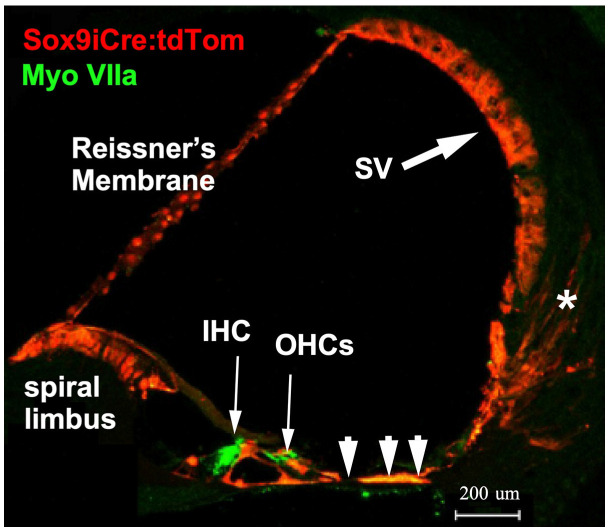
Cre recombinase activity was assessed in the mouse cochlea. Transgenic mice produced to carry a BAC clone containing the promoter region of the Sox9 gene and an ERT2 inducible Cre recombinase were bred with the Ai14 tdTomato mouse line. Tamoxifen treatment activates the Cre recombinase that then removes the stop codons in front of the tdTomato reporter sequence. All cells with tamoxifen-activated Cre recombinase then express the red fluorescent tdTomato reporter gene. In the adult cochlea, cells that harbor activatable Cre recombinase include interdental cells and inner sulcus cells (poorly visible here) of the spiral limbus, pillar cells and Deiter’s cells, supporting cells lateral to the outer hair cells (arrowheads), cells of the spiral prominence, cells of the stria vascularis (SV, large arrow), and a scattering of Type II fibrocytes of the spiral ligament (asterisk).

Sox9 is expressed in pro-sensory regions during early development of the inner ear, but by E14.5, expression is limited to supporting cells [41]. To reveal the cells that functionally express Sox9-driven inducible Cre recombinase (iCre) in the cochlea, mice carrying a Sox9i BAC fused with iCre were crossed with ROSA26 tdTomato reporter mice (Ai14) harboring a floxed stop codon ahead of the tdTomato gene to generate double transgenic mice (Sox9iCre:tdTomato). To ensure that only the expected adult-stage cochlear-supporting cells express the iCre recombinase and therefore would harbor any targeted gene ablation, only adult (2–4 month-old) progeny were injected with tamoxifen to induce tdTomato expression. Immunostaining for red fluorescent protein (RFP, tdTomato) revealed that tamoxifen activated Sox9iCre and induced tdTomato expression in numerous supporting cell types of the adult cochlea (Figure 1). The tdTomato red fluorescent reporter was present in Reissner’s membrane, epithelial-supporting cells lining the cochlear duct, including cells within the inner and outer sulcus, and to a lesser degree, within the type-IV fibrocyte region, and portions of the stria vascularis. The distribution of tdTomato fluorescence (an indication of tamoxifen activatable Sox9iCre) in the mature mouse cochlea was consistent with previous assessments of Sox9 mRNA expression. tdTomato expression was limited to supporting cells and was not expressed by hair cells or spiral ganglion cell neurons [37,41] (and see Figure 4f in [38]). The RFP-positive cells therefore represent the full set of cells that could undergo inducible recombination of any floxed gene of interest. The final subset of these RFP-positive cells that could express a functional phenotype resulting from the genetic ablation of floxed exons of our genes of interest are only those cells lining the scala media that also express GR or MR (i.e., those cells expressing both Sox9iCre and either floxed Nr3c1 (GR) or Nr3c2 (MR)). A number of studies have localized GR and MR along the cochlear spiral using immunohistochemical [3,42], in situ hybridization [43], autoradiographic binding [44], and traditional biochemical binding [45,46] methods. MR and GR seem to be co-expressed in many classes of supporting cell, including supporting cells: (1) at the top of the spiral limbus, including the interdental cells; (2) lining the spiral limbus inner sulcus; (3) leading from the inner sulcus to the inner hair cells; (4) of the outer sulcus, which range from the region lateral to the outer hair cells to the spiral prominence (inclusive of these cells); as well as (5) the pillar cells, (6) Deiter’s cells, and (7) Henson’s cells. Using immunostaining, Kil and Kalinec also report GR expression in Type I, III, and IV fibrocytes of the lateral wall, while MR expression was localized in some cells of the stria vascularis [3]. However, earlier work reports a heavier expression of MR in the stria vascularis [42,43,44].

### 2.2. Validation of Floxed GR and Floxed MR Mouse Lines for Manipulation of Nr3c1 and Nr3c2

We assessed the overall efficiency of the tamoxifen-driven recombination event in the cochlea at the floxed Nr3c1 and floxed Nr3c2 gene exon 2 via a qRT-PCR approach. We adhered to MIQE guidelines [47] for qRT-PCR, specifically assessment of primers for intra- and intermolecular disqualifications (presence of hairpin loops, primer dimers, etc.), validation of amplification efficiency, fidelity of amplification, and use of multiple reference genes for assessing ∆∆Ct-based target gene expression level changes. To validate the qRT-PCR experimental design, primers to be used for cochlear expression studies were first tested on cDNA generated from total RNA isolated from mouse brain. Brain cDNA was used as template for primer validation purposes because of the low recovery rates of total RNA from the mouse cochleae samples. All designed primers amplified targets between 20 and 26 cycles (average approximately 24 cycles, Supplementary Materials Figure S1). The cycle number required to reach threshold has been shown to be a good predictor of amplification of desired target or artifact, with only 5% of reactions with a Ct under 27 producing spurious amplification [48]. This suggests that amplicons are produced from the desired target. Further, melt curves were produced for amplicons of all primer sets at the end of the PCR (Supplementary Materials Figure S2). Melt curves showed a single peak, indicating a homogeneous amplicon population. Finally, PCR amplification efficiency was assessed. A three-step serial dilution of total brain cDNA was used for assessing amplification efficiency of all primers. Amplification efficiency (Supplementary Materials Figure S3) of all primers ranged between 108% and 111% with an R^2^ of 0.977 to 1.000. The outcomes of these preliminary tests indicate high confidence that the primer designs and isolation of high-quality RNA are excellent components of a qRT-PCR experimentation panel. These results were used to select the best primer sets for each gene of interest for use on the cochlear samples.

**Figure 2:**
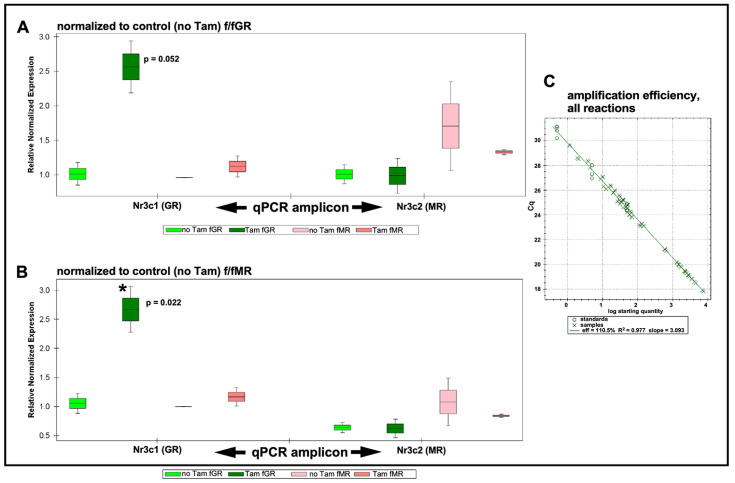
qRT-PCR results. Expression levels are grouped by target gene as assessed in each mouse line and with either the GR or MR serving as normalizer. (**A**) qRT-PCR results are normalized to the control (no tamoxifen) expression level of GR. Following tamoxifen treatment, Nr3c1 (GR) expression (green bars) is increased approximately 2.6×. When normalizing to GR this trend lies just outside standard statistical significance (*p* = 0.052), but when normalizing to MR, this increased GR expression is significant (*p* = 0.022). Expression of Nr3c2 is slightly elevated (pink bars) when normalized to Nr3c1 control (no tamoxifen). Expression of Nr3c2 is slightly (approximately 16%) decreased following tamoxifen treatment. No change was observed in Nr3c1 expression level under these conditions. (**B**) Similar trends as described when normalizing results to Nr3c1 in (**A**) were observed when normalizing data to Nr3c2. Up-regulation of Nr3c1 was statistically significant (asterisk). (**C**) The amplification efficiency for all sample reactions (X symbols), including reference genes (circles), is plotted. Average amplification efficiency was 110.5%. See Supplementary Materials Figures S4 and S5 for PCR subject group design and analysis plots of individual biological repeats. Boxes indicate mean with upper and lower quartiles; whiskers indicate SEM.

Total cochlear RNA was extracted from floxed GR and floxed MR mice that had undergone intraperitoneal (IP) tamoxifen injections for 5 consecutive days (75 mg/kg/day). One week following the last tamoxifen injection, cochleae were isolated and pooled into groups as described in the Methods section. Total RNA was isolated and validated for integrity and purity as described in the Methods section. cDNA was prepared and used for qRT-PCR analysis of tamoxifen-driven alterations to expression levels of GR and MR in both homozygous floxed mouse lines. Expression data were then plotted normalized to the control (no tamoxifen) f/fGR when assessing expression levels of GR and MR in tamoxifen-treated f/fGR mice (Figure 2A). Similarly, expression data were plotted normalized to the control (no tamoxifen) f/fMR when assessing expression levels of GR and MR in tamoxifen-treated f/fMR mice (Figure 2B). In the tamoxifen-treated floxed MR mouse line, qRT-PCR analysis revealed a knockdown of Nr3c2 (the MR gene) by approximately 16% relative to the control (no tamoxifen injection) mice. A slight increase (approximately 10%) was observed in the expression level of Nr3c1 (the GR gene) in tamoxifen-treated f/fMR mice. Surprisingly, tamoxifen treatment of the floxed GR mice resulted in a 260% increase in Nr3c1 expression relative to baseline (no tamoxifen treatment) conditions. Analysis revealed that this increase was just outside of standard definitions for statistical significance (*p* = 0.052) when normalized to GR baseline. While perhaps of limited interpretive use, when normalizing to MR baseline (no tamoxifen treatment), this GR over-expression does reach significance (*p* = 0.022). There was no change to Nr3c2 expression in tamoxifen-treated f/fGR mice. The amplification efficiency of all reactions was monitored (Figure 2C) and falls within the acceptable range such that the ∆∆Ct procedure for quantification of expression level changes can be used. We noted tight variances for some samples. These observations were traced to our use of a compound reference gene set (beta-actin and GAPDH) whose arithmetic mean was used as a comparator for differential gene expression analysis. See Methods for further discussion. Only small changes to either Nr3c1 or Nr3c2 expression levels should be expected following tamoxifen treatment given that the number of cells undergoing tamoxifen-induced recombination events is small compared to the total number of cells in the cochlea. Thus, the tamoxifen-induced events should be thought of from a global cochlear perspective as a transcriptional knock-down of each target gene in the targeted cell population only. Overall, these results demonstrate that tamoxifen treatments of adult floxed GR and MR alter the expression levels of the genes of interest. These procedures can therefore be used to further explore the roles of these genes in the targeted cell populations of the cochlea.

### 2.3. MR and GR Ablation Effects on Auditory Physiology

#### 2.3.1. Supporting Cell MR Ablation Alters Cochlear Physiological Response to Noise

**Figure 3:**
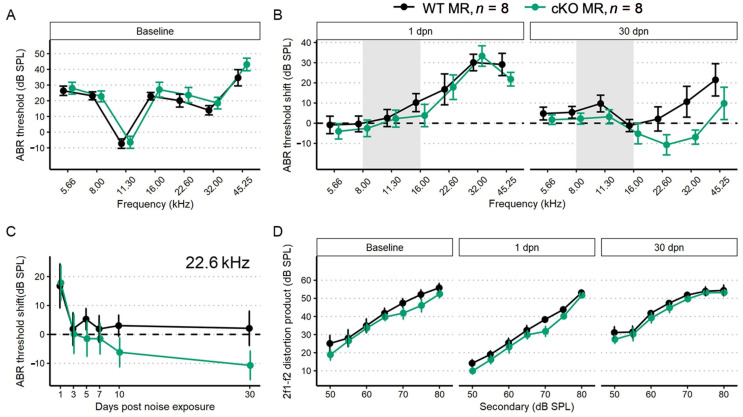
ABR threshold and DP amplitudes (mean ± SEM) from five-month-old mice (*n* = 8 each group) after supporting cell MR ablation and 100 dB SPL mild noise exposure. (**A**) Baseline ABR threshold frequency tuning curve. Data from cKO MR mice are green. Ablation of MR did not result in a significant change to baseline thresholds. (**B**) ABR threshold shift plots for 1 and 30 dpn. A threshold shift of zero indicates a full return to pre-noise threshold and would be indicated by a plot along the dashed line. While at 1 dpn there was no difference in threshold shift between MR control (no tamoxifen) and cKO mice, by 30 dpn, cKO mice demonstrated a trend toward more sensitive ABR thresholds at frequencies mapped basal of the region directly impacted by noise. (**C**) ABR threshold shift at 22.6 kHz plotted against days post noise exposure clearly demonstrates the temporal dynamics leading to increased threshold sensitivity. (**D**) DP amplitudes with f2 = 22.6 kHz sound stimulus at baseline, 1, and 30 dpn did not change between groups, suggesting that changes in mechanics of the basilar membrane may not produce the increased threshold sensitivity shown in (**B**,**C**). Two-way ANOVA tests were used for statistical assessments with statistical significance assigned to *p* < 0.05.

We performed a battery of auditory physiology assessments to investigate the influence of supporting cell MR expression on peripheral auditory function at rest and during recovery from 100 dB SPL, 8–16 kHz noise exposure in adult mice (5 months old at start of study). Physiological assessments included recordings of distortion product otoacoustic emission (DPOAE) amplitudes and pure-tone auditory brainstem response (ABR) thresholds and threshold shifts (Figure 3A–D). DPOAEs and ABRs were recorded at baseline and on days 1, 3, 5, 7, 10 and 30 post-noise exposure. Floxed MR mice received either corn oil (control (no tamoxifen)) or tamoxifen (conditional knockouts, cKOs) injections (75 mg/kg IP 1×/day for 5 consecutive days). Auditory physiological recordings were completed for control (no tamoxifen) MR mice (*n* = 8: 5 males; 3 females) and cKO MR mice (*n* = 8: 5 males; 3 females). At baseline (Figure 3A), there were no differences in ABR thresholds. Day 1 post noise exposure (dpn) and 30 dpn were selected as times representative of the greatest temporary threshold shift (day 1 threshold minus baseline threshold is day 1 compound threshold shift; day 1 compound threshold shift minus day 30 threshold is defined as the maximal (day 1) temporary threshold shift) and permanent threshold shift (defined as threshold on day 30 minus baseline threshold) (Mills 1973; Miller 1974). One day following noise exposure, both groups exhibited a maximum threshold shift of approximately 30 dB SPL at 22.6 kHz and 32 kHz. There were no differences in noise-induced ABR threshold shifts across the tested frequencies between the groups at 1 dpn (Figure 3B). At 30 dpn, however, frequency-specific permanent threshold shifts appeared slightly elevated in control (no tamoxifen) MR mice at 32 kHz (10 dB SPL) and 45.25 kHz (20 dB SPL) while the cKO MR mice exhibited not only a return toward baseline but appeared improved in cKO MR mice. At 22.6 kHz and 32 kHz, thresholds had improved by approximately 10 dB SPL over baseline. While a permanent threshold shift persisted at 45.25 kHz, the threshold shift was approximately 10 dB SPL better than the permanent shift observed in control (no tamoxifen) mice.

To illustrate recovery dynamics from the 100 dB SPL 8–16 kHz octave band noise exposure, we examined the response properties at 22.6 kHz further (Figure 3C). The 22.6 kHz region shows the highest 1 dpn temporary threshold shift and the most dramatic recovery by 30 dpn. Mouse auditory function at 22.6 kHz is relatively sensitive and the half-octave shift phenomenon defines the 22.6 kHz frequency region as the most vulnerable to the 8–16 kHz noise band [49]. Recovery dynamics were virtually identical between control (no tamoxifen) and cKO mice for the first 3 days. Temporary threshold shifts maximally recovered within the first 3 dpn, indicated by the plateau of threshold shift from 5 to 30 dpn. In the MR cKO mice, recovery continued beyond 3 dpn, hitting the baseline value at 5 dpn (control (no tamoxifen) mice never recovered to baseline), remained at approximately the same recovery level through 7 dpn, then underwent an apparent second phase of “enhanced recovery” between 7 and 10 dpn and again further improving between 10 and 30 dpn, hitting a re-established threshold of 10 dB SPL better (more sensitive) than baseline. No plateau was recorded for recovery of the MR cKO 22.6 kHz ABR threshold because the experiment was terminated at 30 dpn. While these data indicated a strong trend for a differential recovery of ABR thresholds between groups, strict statistical significance was not attained. There were no classical outlier data points that could force the data away from statistical significance and variance was similar between groups. The 2f1–f2 distortion product otoacoustic emission with f2 = 22.6 kHz was not statistically different between control (no tamoxifen) and cKO MR mice at baseline or at either 1 dpn or 30 dpn (Figure 3D) as would be expected for the relatively mild noise exposure. Because the 22.6 kHz frequency region is vulnerable to 8–16 kHz noise band, we analyzed this frequency more extensively to assess noise-induced effects. We explored the ABR P1 amplitude and latency to further assess whether MR plays a role in modulating afferent activity following noise exposure.

**Figure 4:**
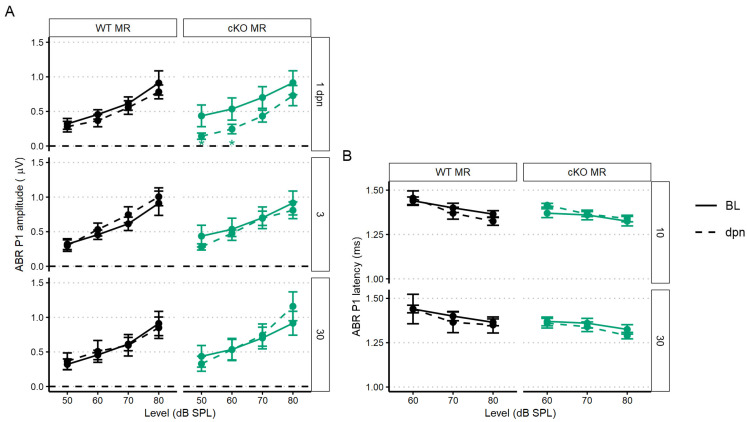
The 22.6 kHz ABR P1 amplitude and latency (mean ± SEM) of cKO MR and control (no tamoxifen) mice following 100 dB SPL mild noise exposure. (**A**) ABR P1 amplitude at 1, 3, and 30 dpn is indicated by a dashed line. Baseline P1 amplitudes are indicated by a solid line (BL). P1 amplitudes were suppressed 1 dpn in the MR cKO mice, but this rapidly recovered to baseline by 3 dpn. No effects of 100 dB SPL noise were observed in control (no tamoxifen) mice. (**B**) ABR P1 latency at 10 and 30 dpn is plotted with baseline P1 latency. No noise-induced changes were evident. Linear mixed effects models were performed in R for statistical analysis.

The first peripheral auditory component vulnerable to noise-induced damage and loss are the IHC-SGN afferent fibers. The ABR P1 amplitudes and latencies are indicators of increasing afferent fiber recruitment and coordinated neural response, respectively, with increasing sound stimulus [50]. To assess the compound neural response generated by ABR stimulation with 22.6 kHz sound, we used linear mixed-effects models: the P1 amplitude model included all recordings (baseline, 1, 3, 5, 7, 10, and 30 dpn) and the P1 latency model included recordings at baseline, 10 and 30 dpn. Noise drove a significant reduction in P1 amplitude at 1 dpn in MR cKO mice which recovered to baseline amplitudes by 3 dpn (Figure 4A). Neither P1 amplitude nor P1 latency was reduced at later times (Figure 4A,B).

#### 2.3.2. Supporting Cell GR Ablation Enhances ABR Threshold Sensitivity but Impairs ABR Threshold Recovery after Mild Intensity Noise Exposure

**Figure 5:**
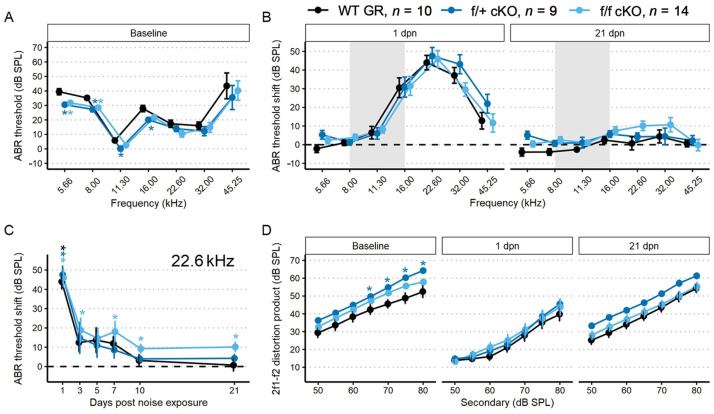
ABR threshold and DP amplitudes (mean ± SEM) were measured in 2–4-month-old mice (*n* = 33) after supporting cell GR ablation and 94 dB SPL mild noise exposure. (**A**) Baseline ABR thresholds from low-to-high recording frequency. Data from f/+ cKO GR mice are blue and f/f cKO GR mice are light blue. Ablation of GR resulted in a decrease of ABR thresholds. (**B**) ABR threshold shift plots following 94 dB SPL exposures at 1 and 21 dpn. The grey bar indicates 94 dB SPL exposing noise bandwidth. Baseline at zero is indicated by a dashed line. While all mice recover from the noise-induced TTS, the f/f mice do not regain baseline sensitivity except at 45.25 kHz. (**C**) ABR threshold shift with 22.6 kHz sound stimulus along dpn, demonstrating lack of full threshold recovery of tamoxifen-treated f/f mice. (**D**) DP amplitudes with f2 = 22.6 kHz sound stimulus at baseline, 1, and 21 dpn. At 1 dpn, all groups show a depressed 2f1–f2 amplitude at 22.6 kHz. While a trend occurs with f/+ mice recovering slightly more amplitude than control (no tamoxifen) or f/f mice, no statistical significance was found. Overall, recovery of DP amplitudes by 21 dpn nearly returned to baseline values, with some small permanent loss at the lowest sound intensities. Asterisks indicate *p* < 0.05 difference to control (no tamoxifen). In all panels, the color-coded asterisks are coupled with the similarly color-coded data for each genotype group (plotted along each colored line) and indicate significant difference from control (no tamoxifen) of that group at *p* < 0.05. Two-way ANOVA tests were used for statistical assessments with statistical significance assigned to *p* < 0.05.

Tamoxifen (or corn oil vehicle for controls) was administered to heterozygous (f/+) and homozygous (f/f) floxed GR:Sox9iCre mice (all 2–4 months old) as described for the MR cKO mice. Auditory physiological recordings were completed for control (no tamoxifen) GR mice (*n* = 10: 6 males; 4 females), and for tamoxifen-treated f/+ cKO GR mice (*n* = 9: 5 males; 4 females), and f/f cKO GR mice (*n* = 14: 10 males; 4 females). Baseline ABR thresholds were obtained after tamoxifen or corn oil injections. In f/+ and f/f cKO GR mice, baseline ABR thresholds were 5–10 dB SPL lower at low frequencies (5.66–16 kHz) compared to control (no tamoxifen) GR mice (Figure 5A). Larger DPOAE amplitudes were also detected with f2 = 22.6 kHz for f/+ cKO GR mice, and trended larger for f/f cKO GR mice, compared to control (no tamoxifen) GR mice (Figure 5D). Across the remainder of f2 frequencies at baseline, no other DPOAE amplitudes were larger than those in control (no tamoxifen) GR mice.

To assess recovery from mild noise exposure, mice were exposed to 94 dB SPL, 8–16 kHz sound for two hours. There were similar ABR threshold shifts across groups at 1 and 21 dpn. We chose to examine the temporal dynamics of recovery from noise exposure in more detail by looking at the 22.6 kHz threshold for two reasons: (1) this is the frequency region primarily affected by the 8–16 kHz octave band noise [51]; and (2) this is a frequency that normally has low thresholds and relatively large neural representation and therefore has the greatest range for detecting and quantifying potential threshold shifts and suprathreshold activity changes.

Examining baseline-normalized ABR threshold shifts revealed that all groups of mice experienced an approximately 45 dB SPL threshold shift at 1 dpn (Figure 5B). Control (no tamoxifen) and f/+ cKO GR mice recovered to baseline ABR values by 10 dpn while f/f cKO GR mice did not recover to baseline ABR threshold values by 21 dpn and maintained a significant threshold shift of approximately 18 dB SPL at 7 dpn and 10 dB SPL at 10, and 21 dpn (Figure 5C). Thus, conditional ablation of both GR alleles from Sox9^+^-supporting cells induced greater vulnerability to permanent noise-induced threshold shift at 22.6 kHz following a noise exposure that did not induce a permanent shift in control (no tamoxifen) or f/+ tamoxifen-treated (heterozygous cKO GR) mice. We further analyzed ABR P1 amplitudes and latencies to characterize noise vulnerability.

**Figure 6:**
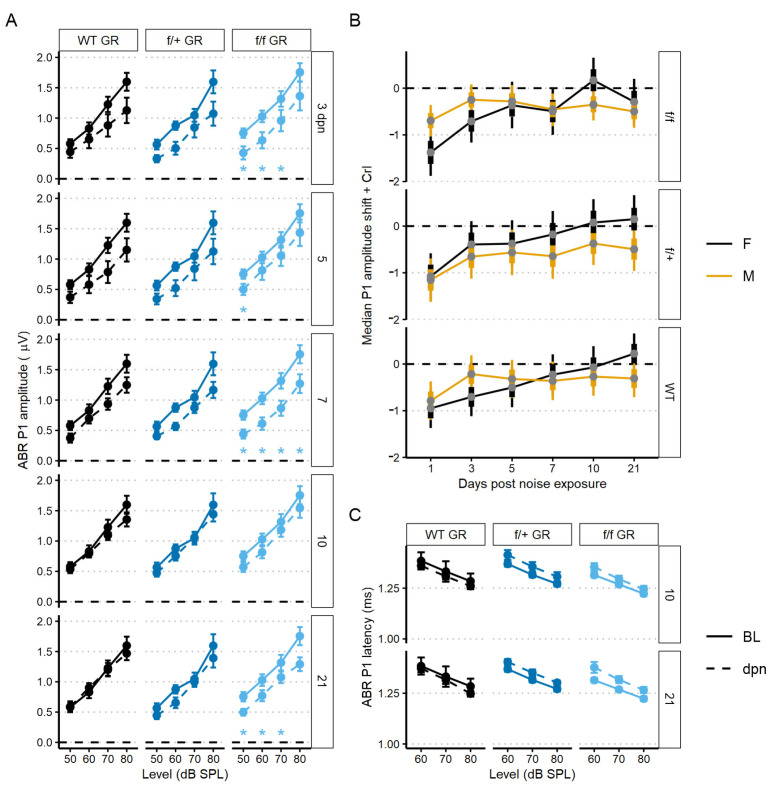
The 22.6 kHz ABR P1 amplitude and latency (mean ± SEM) of GR cKO and control (no tamoxifen) mice following 94 dB SPL mild noise exposure. (**A**) ABR P1 amplitude at 3, 5, 7, 10, and 21 dpn is the dashed line. Baseline P1 amplitudes are indicated by a solid line. Plots are grouped column-wise by genotype and row-wise by dpn. P1 amplitudes were suppressed in f/f cKO GR mice at all dpn, although the suppression was less at 10 dpn before increasing again by 21 dpn. (**B**) Resampled P1 amplitude shift with 22.6 kHz and 80 dB SPL sound stimulus. Plots are grouped row-wise by genotype. Male mice are indicated in yellow. (**C**) ABR P1 latency at 10 and 21 dpn is plotted with baseline P1 latency. No noise-induced changes were evident. Statistical analyses included use of linear mixed effects models for data plotted in A and C and Bayesian repeated measures for B.

To assess recovery dynamics in ABR P1 amplitudes, we used a linear mixed-effects model to analyze level-driven P1 amplitudes at 22.6 kHz sound stimulus with baseline, 3, 5, 7, 10 and 21 dpn. Multiple comparisons to baseline values revealed that both control (no tamoxifen) and f/+ tamoxifen-treated (heterozygous cKO GR) mice recovered to pre-noise P1 amplitudes driven by 50–80 dB SPL sound intensities. Across control (no tamoxifen) and f/+ tamoxifen-treated (heterozygous cKO GR) mice, amplitudes were altered by noise from 3 to 7 dpn but were already statistically similar to baseline followed by appearance of a complete recovery by 10 dpn. However, f/f cKO GR mice had incomplete recovery to baseline P1 amplitudes. f/f GR mice presented with significant ABR P1 reduction at 3, 7, and then persistently at 21 dpn (Figure 6A). A trend toward slightly longer latencies was observed in both the tamoxifen-treated f/+ and f/f mice compared to control (no tamoxifen) mice, but this did not reach the level of statistical significance (Figure 6C).

We next sought to investigate potential sex effects related to GR functionality. We modeled sex effects on 80 dB sound stimulus-driven 22.6 kHz ABR P1 amplitudes using a Bayesian inferential framework to improve detection of mild injury types [52]. Sex differences were supported for the f/f cKO GR noise-vulnerable group. Specifically, male mice had less P1 reduction at 1 dpn compared to the females (Bayes Factor [BF_10_] = 3.2), but there was evidence to support a moderate P1 amplitude decrease at 21 dpn in male mice when compared to baseline (BF_10_ = 4.4, 95% CrI = −0.85 to −0.16) (Figure 6B, 0 value = dashed line). In contrast, at 21 dpn, female f/f cKO GR mice have P1 amplitude shift intervals which overlap zero (BF_10_ = 0.4, 95% credible interval [CrI] = −0.8 to 0.2). While support was diminished for f/+ heterozygous cKO GR male mice, credible intervals suggest relatively greater support for P1 amplitude loss (BF_10_ = 0.9, 95% CrI = −0.96 to −0.02). For control (no tamoxifen) GR male mice, credible intervals suggest near complete recovery of P1 amplitudes (BF_10_ = 0.4, 95% CrI = −0.71 to 0.1). GR-related male noise vulnerability is consistent with reports of male susceptibility to sensory and neural hearing losses compared to noise susceptibility of females [53,54,55].

### 2.4. Effects of GR and MR Ablation on Afferent Synapse Innervation to Inner Hair Cells

**Figure 7:**
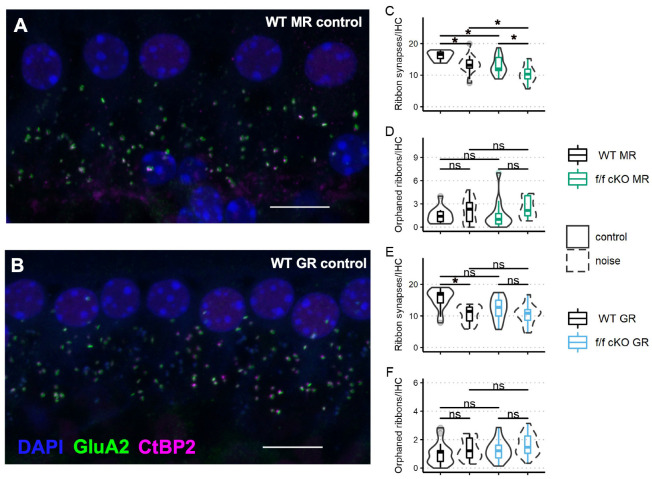
Ribbon synapse and orphaned ribbon counts per inner hair cell in the approximate 22 kHz region from mice at 21 dpn and from age-matched controls. Representative images of inner hair cell ribbon synapse staining from a control (no tamoxifen) MR mouse (**A**) and a control (no tamoxifen) GR mouse (**B**) are shown. (**C**) Paired CtBP2-GluA2 counts averaged over inner hair cells imaged from fMR:Sox9iCre mice. (**D**) Orphaned CtBP2 counts per inner hair cell averaged over inner hair cells imaged from fMR:Sox9iCre mice. (**E**) Paired CtBP2-GluA2 counts averaged over inner hair cells imaged from fGR:Sox9iCre mice. (**F**) Orphaned CtBP2 counts per inner hair cell averaged over inner hair cells imaged from fGR:Sox9iCre mice. Scale bar = 10 μm. Magenta: CtBP2, green: GluA2, blue: DAPI. Linear mixed effects models were employed for data plotted in C-F. Multiple comparisons were with Tukey correction. Significance (*) is *p* < 0.05 and ns indicates “not significant”.

Next, we assessed ribbon synapse counts and orphaned ribbon counts per inner hair cell as a morphological correlate of afferent synaptopathy. C-terminal binding protein 2 (CtBP2) is a constitutive protein of the presynaptic “ribbon” release site in photoreceptor cells and hair cells and some specialized neurons within the brain. The glutamate receptor AMPA subunit 2 (GluA2) controls calcium permeability through AMPA-responsive glutamate receptors and is present in all adult AMPA glutamate receptor patches on SGN dendrites [56]. Paired (directly opposing) CtBP2-GluA2 immunolabelled patches are indicative of a functional afferent synapse between the hair cells and ganglion cells. We immunostained cochlear whole mount tissue of f/f MR control (no tamoxifen) mice (without noise: *n* = 3 mice [2 males; 1 female] and plus noise: *n* = 5 mice [2 males; 3 females]) and f/f MR cKO mice (without noise: *n* = 4 mice [4 males] and plus noise: *n* = 4 mice [4 males]) (Figure 7A) with antibodies to CtBP2 and GluA2 (Figure 7A,B) and then counted CtBP2-GluA2 pairings as a measure of afferent synapse integrity (Figure 7C,D). We chose the 22.6 kHz cochlear region for analysis because this region is predicted to be one of the most affected by an 8–16 kHz noise band [49,57,58,59] and is ideal to assess noise-induced damage. We analyzed the average number of synapses per image with a linear mixed-effects model. We identified a significant effect of noise as driving ribbon synapse loss in control (no tamoxifen) MR mice. In cKO mice, in which supporting cell MR was ablated, ribbon synapse numbers were decreased even prior to noise (Figure 7C). The median number of averaged CtBP2-GluA2 opposed profiles was 12 per IHC, roughly similar to the median number of intact synapses of control (no tamoxifen) mice that experienced the 2 h, 100 dB SPL sound exposure (13 per IHC). Interestingly, cKO MR mice experienced only marginally greater noise-induced ribbon synapse loss beyond that observed in the cKO mice that did not experience noise exposure (Figure 7C). Prior research has shown that orphaned ribbons, defined as CtBP2 profiles not opposed by postsynaptic GluA2 profiles, are increased immediately after noise exposure but are resolved by 7 dpn [60,61]. We examined afferent synapse integrity in mice that were recovered to at least 30 dpn, our experiment endpoint. Our analyses of orphaned ribbons at 30 dpn showed no differences among wild-type and cKO mice in both the noise-exposed and control animals (Figure 7D). In a similar manner, we analyzed ribbon synapses in fGR:Sox9iCre mice (control (no tamoxifen) without noise: *n* = 5 mice [3 males; 2 females], control (no tamoxifen) plus noise: *n* = 4 mice [2 males; 2 females], cKO without noise: *n* = 5 mice [3 males; 2 females], cKO plus noise: *n* = 6 [4 males; 2 females]) (Figure 7B,E,F) to asses noise-induced loss of ribbon synapses (afferent pre- and post-synaptic elements). Analysis of ribbon synapse counts revealed similar ribbon synapse vulnerability to mild 94 dB SPL noise exposure across control (no tamoxifen) and f/f cKO GR mice (Figure 7E). Noise-exposed control (no tamoxifen) GR mice had reduced ribbon synapse counts compared to age-matched control (no tamoxifen) no-noise. A trend was revealed in which noise-exposed cKO GR mice had reduced ribbon synapse counts relative to age-matched no-noise cKO GR mice. Additionally, a trend for cKO GR mice to have reduced ribbon synapse counts under basal conditions might have influenced the final results, producing a non-significant noise-induced ribbon synapse loss (Figure 7E). Neither noise nor expression status of GR in supporting cells altered counts of IHC orphaned ribbons (Figure 7F).

### 2.5. Effects of GR and MR Ablation on Innate Immune Cell Presence near the Organ of Corti under Basal and Noise-Exposure Conditions

Iba1^+^ microglia, macrophage-like glial cells of the CNS, are classically described in one of two morphological phenotypes. Ramified microglia are considered to be surveilling their environment. These cells typically retract their processes and attain an ameoboid shape when a physical injury occurs and in this form function as macrophages, phagocytosing cellular debris. However, while not producing overt pathology, stressful events are also known to impact microglial morphology. Previous work has demonstrated that under chronic stress conditions, Iba1^+^ microglia do not transform to the ameoboid morphology, but rather increase process ramification [62]. Further, such morphological changes seem not to be in response to CNS injury. Iba1 is a marker of macrophage-like cells in the cochlea [15,17] and noise exposure increases the cochlea’s immune response, resulting in an increase in Iba1^+^ cells.

To investigate whether the noise-induced inflammatory state of the cochlea was altered by MR or GR ablation from Sox9^+^-supporting cells, we counted Iba1^+^ immunolabeled cells in the sensory epithelium at 7 dpn. While Iba1^+^ immunostaining cannot by itself distinguish between resident and infiltrating macrophage-like cells [63,64,65,66], it is still a useful morphologically-based assessment of potential inflammatory processes occurring in response to noise exposure. We chose 7 dpn because previous research has shown innate immune response increases to its greatest levels in the cochlea after intense (105 dB SPL) noise exposures [15,16,64,65,67] between 3 and 7 dpn and might therefore also be an ideal time to detect differences for innate immune cell presence after the relatively mild (100 dB SPL) noise exposures [18,19,68].

**Figure 8:**
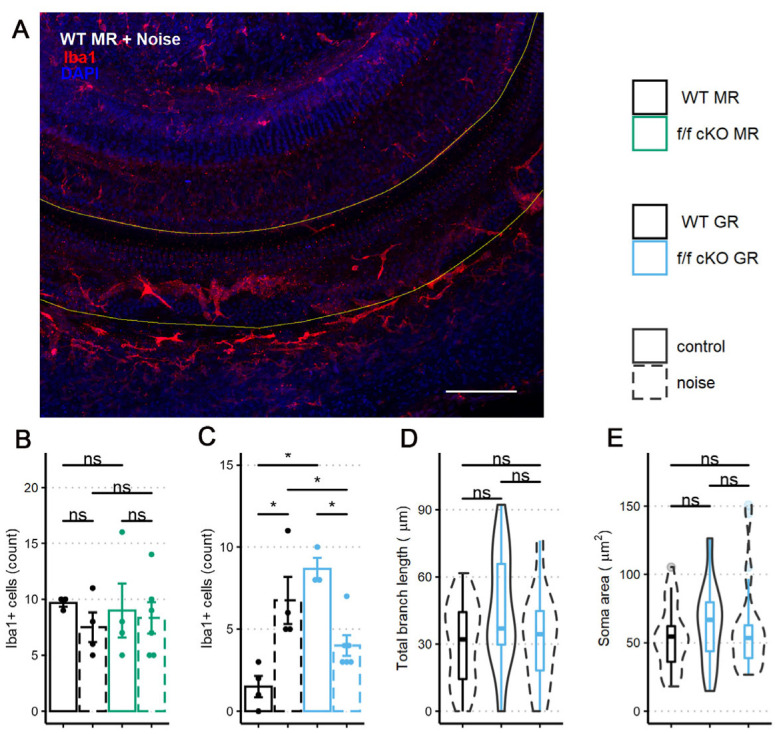
Iba1^+^ cells within the middle-turn sensory epithelium of mice at 7 dpn and from age-matched controls. (**A**) A representative image of a sensory epithelium wholemount prep from a noise-exposed control (no tamoxifen) fMR:Sox9iCre mouse. (**B**) Iba1^+^ cell counts per stretch of the middle turn imaged from fMR:Sox9iCre mice. (**C**) Iba1^+^ cell counts per stretch of the middle turn imaged from fGR:Sox9iCre mice. (**D**) Total branch length of Iba1^+^ cells within the sensory epithelium. (**E**) Iba1^+^ cell soma surface area within the sensory epithelium. Scale bar = 100 µm. Red: Iba1, blue: DAPI. Two-way ANOVA tests were used for statistical assessments of data plotted in B-C and linear mixed effects models were used for data plotted in D-E. Multiple comparisons used the Tukey correction. Significance (*) is *p* < 0.05 and ns indicates “not significant”.

We immunostained wholemount cochlear pieces from control (no tamoxifen) and homozygous (f/f) cKO MR mice with anti-Iba1 antibodies (control (no tamoxifen) without noise: 3 mice [3 males], control (no tamoxifen) with noise: 4 mice [3 males; 1 female], cKO without noise: 4 mice [4 males], cKO with noise: 6 mice [3 males; 3 females]). A representative immunolabeling of the middle-turn sensory epithelium containing the 22.6 kHz region is shown in Figure 8A. The sensory epithelium length (range: 576 to 861 μm) was imaged to ensure that roughly equal areas were assessed among specimens. While control (no tamoxifen) MR mice exhibited nearly identical numbers of Iba1^+^ cells at basal (no noise) conditions, a trend occurred in which the mean number of Iba1^+^ cells decreased and variation between mice increased at 7 dpn (Figure 8B). Under MR cKO conditions, both no noise and noise exposed mice were similar to the control (no tamoxifen) noise exposed group with respect to mean number of cells present and variation between samples.

Similar to the analysis of MR control (no tamoxifen) and cKO mice, we immunostained wholemount cochleae of control (no tamoxifen) and homozygous (f/f) cKO GR mice to quantify the presence of Iba1^+^ cells in the middle-turn sensory epithelium that includes the 22.6 kHz frequency region (control (no tamoxifen) without noise: 4 mice [4 males], control (no tamoxifen) with noise: 4 mice [2 males; 2 females], cKO without noise: 3 mice [3 males], cKO with noise: 6 mice [3 males; 3 females]). Sensory epithelium length was similar between groups (range: 600 to 840 μm). Counts and analysis of Iba1^+^ cells (Figure 8C) revealed an interaction of supporting cell GR expression and noise exposure with the number of Iba1^+^ cells present in the sensory epithelium. GR ablation from Sox9-expressing supporting cells induced a greater number of Iba1^+^ cells in the sensory epithelium under no-noise conditions. Further, noise exposure of cKO GR mice resulted in fewer Iba1^+^ cells at 7 dpn when compared to noise-exposed control (no tamoxifen) GR mice (Figure 8B).

In an effort to further classify the activity state of these cochlear microglia/macrophage-like Iba1^+^ cells, we analyzed the Iba1^+^ cell phenotype of the GR cKO mice by examining total branch length and cell soma area as an indicator of activation [69] similar to what was reported for microglia. Following GR ablation from supporting cells, there was a trend for immune cells under basal (no noise) conditions to have an increased total branch length (Figure 8D). This result is similar to that described for a chronic-stress state [62,70], indicating that loss of basal cochlear GR signaling from Sox9^+^-supporting cells induces a transition to a stress-activated Iba1^+^ cell phenotype and not a phenotype normally associated with physical damage. However, total branch length and soma area were not different between the noise exposed groups (Figure 8D,E), indicating that supporting cell GR expression may influence the number of Iba1^+^ cells in this cochlear subregion but not the activity state (assessed by cell morphology) induced by mild 94 dB SPL noise exposure.

## 3. Discussion

The goal of our study was to assess the role of the major corticosteroid receptors (glucocorticoid and mineralocorticoid receptors) in a subset of epithelial “supporting cells” along the basilar membrane of the scala media during recovery from mild noise exposure in naïve mice. Using conditional genetic manipulations designed to ablate MR or GR in Sox9^+^ epithelial-supporting cells of the mouse cochlea, we assessed the influence of receptor loss spanning acute and long-term recovery from noise exposure. Our genetic approach forgoes surgical manipulations such as adrenalectomy and enabled investigation of cochlear MR and GR expression in defined cell types of intact mice without impacting extra-cochlear receptor expression (e.g., in neurons or circulating myeloid cells). Our study begins an investigation of complementary roles of MR and GR co-expressed in supporting cell populations with a focus on the physiological impacts of noise on cochlear function and on noise-induced inflammatory responses and regulation occurring in the cochlea. We found that supporting cell MR ablation resulted in transient ABR P1 amplitude decrease after mild noise exposure without affecting physiological outcome. Loss of supporting cell GR expression induced vulnerability to mild noise exposure, including slower and incomplete ABR threshold recovery and persistent physiological impairment. Immune cell presence, used as an indicator of the cochlear inflammatory state, revealed a role of supporting cell GR for basal and noise-induced immune cell activity at 7 dpn. Together, the data indicate distinct roles of Cort-sensitive nuclear receptors expressed in epithelial-supporting cells that may impact intercellular communication throughout the cochlear duct.

As with most gene ablation studies, the observable effect after receptor ablation could be complex and include some degree of compensation (e.g., [71]), either by overstimulation of the remaining Cort-sensitive receptor in other cells/regions of the cochlea or by upregulating receptor expression in cells that have not undergone the gene recombination event. The latter appears to have occurred following the conditional ablation of the GR from Sox9^+^-supporting cells. Our expression data indicate a significant upregulation of GR following tamoxifen activated Cre recombinase in Sox9^+^-supporting cells. The cells participating in this unexpected upregulation of the GR gene are yet to be identified. Both the MR and GR are thought to exist in two states: a cytosolic state that, once bound by ligand, enters the nucleus and modulates transcriptional activity; and a membrane bound, faster-acting state that exerts its activity over other proteins, including ion channels, and signal cascades. Our work here does not dissociate such functional aspects of these receptors but rather considers receptor involvement in cochlear function in a broader context.

A caveat to cKO manipulation of genes of interest lies in understanding when the gene of interest has reached its lowest expression level, especially when subpopulations of cells in a complex tissue are targeted. One may assay mRNA for the gene of interest, or attempt to assess protein levels, but these have the disadvantage that if the target cell population is modest in size relative to other cells, RNA and/or protein-level changes may either seem modest or, in worst cases, be barely detectable. Thus, understanding the mechanisms that rule RNA and/or protein stability for the targeted gene is critical, but such information is often not available. We used two sources of information to think about this problem for the GR (and assumed the MR may behave similarly). Neuronal and synaptic proteins have been investigated and are thought to have a half-life of 3–14 days [72,73]. Our experimental design (five daily tamoxifen injections, at least 2 days of rest (and sometimes more than 5 days), and more than 2 days following baseline testing before experimentation began) suggests that if our gene products act in a manner similar to other neuronal proteins, expression levels would be within the half-life time frame. A study on GR (and by extension, given that MR functions similarly as a DNA-binding/transcriptional factor, we presume a similar fate for MR) demonstrated that the ubiquitin-proteosome degradation pathway terminates GR signaling and that GR is hyperphosphorylated upon ligand (GC) binding, initiating the degradation program [74]. It is conceivable that as the level of GR expression drops in targeted cells, low levels of GC will bind a greater share of remaining GR, accelerating proteosome-associated degradation of the remaining pool of GR.

### 3.1. Validation of the cKO Mouse Models

The cochlea expresses both GR (Nr3c1) and MR (Nr3c2) based on binding assays (MR: [45], GR: [46]), in situ hybridization (MR: [43], GR: [75] and immunolocalization (MR: [42], GR: [3,76]. We produced cKO mouse lines targeting MR and GR expression by using the Sox9 gene to drive tamoxifen-inducible Cre recombinase in cells of interest—the supporting cells lining the scala media. These cells were targeted because our previous work suggested that this population may be involved in cellular stress-response programs. We validated that the supporting cells of interest expressed functional Cre recombinase following tamoxifen treatment by using the tdTomato reporter mouse bred to the Sox9iCre line. While other cells in the cochlea probably also express MR and GR, it is only those cells that express both the iCre (defined by visualizing the tdTomato reporter and the floxed gene of interest (based on previous reports) that will harbor the MR or GR knockout. The cells with both iCre and floxed alleles are the supporting cells lining the scala media, and it is from manipulation of these cells that the observed phenotypes emanate.

### 3.2. The Influence of Supporting Cell MR Expression on Auditory Functional Recovery

Based on the differential occupancy profile by endogenous GCs in the brain, GRs are thought to mediate negative feedback signals following elevated GC levels produced by, e.g., stress, whereas MRs, via their enhanced GC binding affinity (ten-fold higher) over that of the GRs are thought to be involved with tonic influences of GCs [77,78]. Thus, in the absence of significant levels of aldosterone and stress-elevated levels of Cort, the high-affinity mineralocorticoid receptor is thought to be occupied and activated by basal levels of Cort during periods (i.e., non-stress) of low circulating levels of Cort, reserving GR activation for the highest but transitory levels of Cort. Given their higher affinity for Cort, MRs can therefore modify circadian and inflammatory responses via a direct action of competition for ligand until the MR pool is fully saturated. Additionally, MR activity can dampen GR sensitivity to stress-induced levels of GC via direct MR transcriptional regulation of the heat-shock protein 90-associated co-chaperone FK506-binding protein 51 (FKBP5) [79,80].

A role for MR activity during cochlear inflammatory response has not been previously assessed. After MR ablation in the Sox9^+^ epithelial-supporting cell population, we did not observe an effect for peripheral auditory outcome after mild (100 dB SPL) noise challenge. Instead, in MR cKO mice, ABR P1 amplitude was decreased at day 1 post noise, without apparent alteration to OHC activity. It is possible that MR cKO mice experience an exacerbated decrease in endolymphatic potential (EP) driven by noise, though this possibility was not assessed in these studies. Cochlear MR, especially in the stria vascularis, is implicated in ion homeostasis [11,81,82]. MR expression in supporting cells along the cochlear basilar membrane is likely to be involved in endolymphatic ionic balance where partial loss of MR expression could alter the ionic environment enough to produce the temporary threshold changes observed. Indeed, noise challenge produces a decrease in K^+^ ion concentration [83]. However, other reports suggest no change or an increase in K^+^ ion concentration with the occurrence of noise exposure [84,85]. Noise-induced K^+^ concentration increase was suggested to occur after damage to hair cells which normally flux K^+^ along its electrochemical gradient. In addition, noise exposure induces cellular stress within the ion cycling pathway [27]. The effect of decreased EP is well-known to reduce outer hair cell activity. Inner hair cell and afferent fiber activity is also uniformly reduced upon EP decrease [86]. Indeed, ABR P1 amplitude decrease at 1 dpn appeared consistent with poor EP stabilization. Therefore, we consider it most likely that loss of supporting cell MR expression in the cochlea, combined with noise exposure, has induced an acute, noise-induced effect that temporarily impaired generation of the EP [87]. While further experimentation will be required to assess whether MR activity in cochlear-supporting cells modifies the gap junction protein connexon 43 (Cx43) expression and therefore ion recycling between the cochlear duct and spiral ligament in the cochlea, indications from other tissues such as heart and kidney support the general idea that MR signaling can influence ion distribution. In ventricular cardiomyocytes, the activity of MR and its effects on Cx43 are complex [88]. Activation of MR by aldosterone significantly upregulates Cx43, concomitantly increasing conduction velocity significantly, but overstimulation with higher concentrations of aldosterone decreased Cx43 expression. Similarly, complex phenomena may occur in the cochlea. Cx43 is required for EP maintenance and normal auditory function [89].

At the morphological level, supporting cell MR ablation induced ribbon synapse loss. Chronic EP reduction may contribute to age-related hearing loss (ARHL). Auditory neuropathy appeared to be the first sensorineural morphological loss in human and rodent ARHL [90,91,92,93]. Recent investigation demonstrated increased K^+^ concentration in culture media can induce ribbon synapse loss in mouse cochlear explants [94]. However, ribbon synapse loss with supporting cell MR ablation and noise exposure was not exacerbated. Dysregulation of the inflammatory microenvironment may contribute to synaptopathy; pathological levels of TNF*α* perfused into guinea pig cochleae resulted in auditory afferent loss [95], and the innate immune cell response to noise exposure is implicated in ribbon synaptic regeneration [19]. A representative immune response in the sensory epithelium did not associate noise exposure or supporting cell MR with innate immune cell presence. Altogether, the data from fMR:Sox9iCre mice support the idea of a role for supporting cell MR expression in ionic balance regulation rather than modulation of a noise-induced inflammatory and innate immune response.

### 3.3. The Influence of Supporting Cell GR Expression on Auditory Functional Recovery

Glucocorticoids are implicated in modulation of auditory sensitivity. Adrenalectomy, synthetic GCs, and stress exposure can each alter auditory sensitivity [13]. Here, cKO of Sox9^+^-supporting cell GR expression of either one (f/+ mice) or both (f/f mice) alleles induced lower baseline ABR thresholds, especially of low frequencies. These changes to auditory afferent sensitivity are presumably related to greater otoacoustic emission amplitudes across frequencies similar to that produced by f2 = 22.6 kHz and 50–80 dB SPL. Our analysis of noise exposure focused on the 22.6 kHz region because this region is simultaneously sensitive (so it has a low threshold and thus maximal range for quantifying threshold shifts before any ceiling effect truncates data collection) and is one of the most affected regions following exposure to an 8–16 kHz noise band. The 22.6 kHz region is therefore useful for detecting degradation of auditory thresholds after noise-induced damage. While in control (no tamoxifen) and f/f cKO GR mice, DP amplitudes recovered by 21 dpn, the DP amplitudes of f/+ heterozygous cKO GR mice did not recover. ABR thresholds and P1 amplitudes recovered to wild-type values for both control (no tamoxifen) and f/+ heterozygous cKO GR mice. However, the f/f cKO GR mice never regained baseline ABR threshold sensitivity or P1 amplitudes. GR-related physiological vulnerability of f/f cKO GR mice did not relate to increased auditory sensitivity. Indeed, similar ABR threshold shift across groups at 1 dpn (see Figure 6B) suggests that the degree of noise induced dysfunction was not related to an increased initial threshold shift [96] and thus any change in dynamic recovery between the GR mouse lines is not explained by an initially greater vulnerability to noise-induced damage. Overall, though we have targeted a range of supporting cell types, an effect on auditory sensitivity could be through Deiter’s cell modulation of outer hair cell electromotility [97] but appeared independent of vulnerability to noise exposure.

GR ablation from Sox9^+^-supporting cells resulted in delayed and incomplete recovery to baseline ABR threshold and long-term decrease of ABR P1 amplitude after mild (94 dB SPL) noise exposure. Interestingly, auditory physiological impairment was not associated with ribbon synapse loss. However, vulnerability to noise-induced changes in physiology appeared related to the presence of Iba1^+^ immune cells in the sensory epithelium in two ways: GR ablation (1) increased the basal number of immune cells, yet (2) decreased the number of noise-induced immune cells compared to noise-exposed control (no tamoxifen) mice. The first observation appears similar to GR-related regulation of innate immune cell presence near the sensory epithelium [28]. Secondly, we expected supporting cell GR ablation to positively feedback on proinflammatory signaling and exacerbate the immune response. Inflammatory and immune response magnitude driven by noise exposure associates with ribbon synapse repair [19] and permanent threshold shift [98]. In the current study, innate immune response assessed as Iba1^+^ cell counts within the sensory epithelium at 7 dpn was not exacerbated, and instead was reduced, despite auditory physiological deficits in f/f cKO GR mice. Noise–dose divergent cochlear immune response was also found in investigations of circulating Cort related to circadian rhythm state, patterning levels of noise-induced damage with auditory physiology [20,99,100]. Some distinctions about the influence of GR expressed in supporting cells may be made: (1) supporting cell GR knockdown (f/+ heterozygous cKO GR), which could represent circadian time (based on available open receptors) distinct from control (no tamoxifen) mice, had typical physiological recovery from noise exposure and (2) supporting cell GR ablation was detrimental to auditory physiological recovery from noise exposure. The latter is in comparison to a protective effect of adrenalectomy on circadian-patterned susceptibility to noise exposure [20]. However, our expression analysis of GR mRNA levels unexpectedly demonstrated that a loss of Sox9^+^-supporting cell GR expression induces a significant (roughly 2.5×) increase in cochlear GR expression overall. The baseline changes to ABR thresholds shown in the current study suggest a cell-type-specific role of cochlear GR activity that interacts with and may dictate the influence of systemic Cort. Though circadian influence of cochlear-supporting cells should be further investigated (e.g., see [25,101]), the data indicate a role of supporting cell GR expression to modify noise-induced damage to auditory function.

Supporting cells may influence auditory physiology through various physiological effects. Sex effects provide insight into potential pathologic mechanisms after supporting cell GR ablation. In the current study, male mice were more susceptible to noise-induced reduction of P1 amplitudes following ablation of genes expressed in cochlear-supporting cells. Our observations of differential sensitivity to damage between male and female mice is consistent with a meta-analysis in which male rodents experienced greater hair cell loss and threshold shift compared to female rodents [102]. Studies have also found male vulnerability to mild noise exposure and P1 amplitude reduction [54,103], though see [53]. Recent investigation suggests that estrogen may directly modulate spiral ganglion neurons [54]. Our current study suggests that a GR-related pathologic mechanism was in part blunted by estrogen signaling. A prominent protective effect of estrogen in the cochlea is through brain-derived neurotrophic factor (BDNF) signaling (reviewed by [104]). However, BDNF signaling is also reported to exacerbate ribbon synapse loss [105,106]. In our experiments, noise-induced synapse loss was not detected after supporting cell GR ablation (see Figure 8). P1 amplitude reduction in female mice at 1 dpn, which was greater than the P1 reduction of male f/f cKO GR mice at 1 dpn as assessed by Bayesian inferential credible intervals, (Figure 6B) suggests noise-induced neural adaptation present in female mice consistent with the possibility of estrogen-associated neural adaptation. Other potential sex-related differences, such as to OHC adaptation [107,108], inflammatory response, or lateral wall function, will have to be explored by further investigation. Importantly, whichever pathologic mechanisms contributed to noise exposure vulnerability in male f/f cKO GR mice appeared shielded in female mice. Investigation of sex effects for cochlear-supporting cell biology is expected to further mechanistic understanding of these cell types, compensatory processes in the cochlea, and the influence of estrogen signaling.

### 3.4. The Role of Supporting Cells for Auditory Homeostasis and Recovery

Supporting cells perform multiple functions to maintain auditory sensitivity. Within the epithelial-supporting cells, specialized functions include ionic balance through connexin expression and mechanical support, glia-like functions through neurotrophic factor expression and release, and maintenance of extracellular glutamate levels by the glutamate–glutamine cycle [87,109,110,111,112]. Dysregulation of supporting cell functions including clearance of extracellular glutamate, maintenance of K^+^ ion concentration, and alterations to neurotrophic factors each induce synapse loss [94,113,114]. Supporting cell populations are stress responsive and have been associated with injury modulation and as sources of secondary damage. Supporting cell-mediated adaptation involves paracrine release of effector molecules [112,115,116,117]. Furthermore, a cochlear HPA-like homeostatic signaling system implicates supporting cells as a local stress-response system [118], which may combat dysfunction via induction of various intra- and extracellular signaling systems following harmful noise levels without recruiting slower systemic stress responses. Indeed, HPA-like signaling systems expressed in other organs have been demonstrated to allow local immunomodulatory response to their own unique stressors without necessitating systemic stress response [119].

A prominent role of HPA-like activity is immunomodulation via its modulated release of GCs and activation of GC-sensitive receptors. Innate immune cell phenotypic differentiation and proinflammatory macrophage clearance of debris is sensitive to GC signaling [120,121,122]. Our studies showed that in the absence of GR expression in cochlear-supporting cells, Iba1^+^ cells of the cochlea at baseline, no noise conditions presented with a hypertrophied ramification morphology that indicated a chronic stress condition was underway. Interestingly, Iba1^+^ immune cells decreased in number after supporting cell GR ablation and noise exposure (see Figure 8). Fewer Iba1^+^ cells (see Figure 8C,D) is consistent with a state mirroring increased Cort signaling. This appeared to increase resolution of the innate immune cell response. Furthermore, we observed a trend in which loss of supporting cell GR expression reduced ribbon synapse counts (Figure 7E). However, given our finding that cochlear GR expression levels significantly increase following GR ablation from Sox9^+^-supporting cells, care must be taken in interpretation of results. Further work will be necessary to determine where (cell types, regions within the cochlea, etc.) and how this compensation occurs.

Long-term GC signaling can induce glutamatergic excitotoxicity [123,124]. However, GR ablation from Sox9^+^ support cells, which induced a significant compensatory response that increased GR expression 2.5× over baseline levels in other cells, was not associated with noise-induced ribbon loss. Given that prolonged stress and higher circulating Cort is apparently involved with greater noise-induced ribbon synapse loss and spiral ganglion neuron loss [13,125,126], the GR expression compensation observed in our experiments may not reflect higher GR signaling.

Our study represents a first attempt at uncovering the functional role of the GR and MR in distinct cell populations of the cochlea. Further, our approach allowed for the first time the manipulation of GC (Cort and aldosterone) signaling in the cochlea while leaving intact these systemic signaling systems. Despite the advanced methods used to accomplish these tasks, limitations in both experimental design and in interpretation exist. First, in addressing immunomodulatory roles for GR and MR, we assessed Iba1^+^ cell numbers at pre-noise exposure and, importantly, a single discrete post-noise time (day 7) as a metric for inflammatory/immune state responses. However, we have not conducted experiments to assess the more acute phase of inflammatory response, nor longer-term changes, thus leaving open the question of dynamic response of the local immune response to noise under condition of cell-specific MR and GR ablation. Further, our stimuli were relatively mild in this study. Indeed, most studies of the noise-induced cochlear immune response use intense noise exposures and immune cell analysis at day 7. Our results may not represent a maximally recruited immune response with 94 dB SPL noise, but nonetheless are important since most subjects are exposed to less intense noise intensities that arguably do not induce physical damage (hair cell death, membrane rupture, etc.) that can be produced by the higher intensity noise levels used in other studies. Second, our morphological assessment of cochlear damage was primarily through ribbon synapse counts without assessing rearrangement [127] or puncta ultrastructure. The noise exposures used were not expected to impact outer hair cell survival. Assessment of the OHC region by a simple DAPI stain in fact did not reveal any significant loss of OHCs (data not shown). While the ABR P1 amplitudes decreased after noise, amplitudes recovered despite structural synaptopathy based on counts of GluA2/ribbon pairing (see Figure 3, Figure 4, Figure 7 and Figure 8). Synaptopathy without amplitude reduction in the cKO MR loss could be further related to afferent fiber diversity [128] and differential vulnerability to damage/loss.

Overall, this study used a genetic approach to produce a cell-specific, temporally defined conditional ablation of GC receptors that together participate in the basal and stress-related physiological state of the cochlea. Importantly, our mouse models allowed us to separate cochlear MR and GR function from each other while also maintaining normal MR and GR activity outside of the cochlea. Our work demonstrated differential roles of MR and GR during recovery from noise exposure. Sox9^+^-supporting cell GR influenced the susceptibility to permanent NIHL and the rate of auditory functional recovery and resulted in a decrease of ABR P1 amplitudes over the entire time frame examined. The fact that the tamoxifen-treated f/+ heterozygous cKO GR mice mirrored the control (no tamoxifen) mice in both general ABR thresholds and P1 amplitudes further suggests that one allele of GR is sufficient to drive normal GR-based processing in the cochlea. While there was not good evidence that loss of MR from Sox9^+^-supporting cells influences the early dynamics of threshold recovery following noise exposure, our data clearly showed a trend in recovery from 10 to 30 dpn that ended with a state of hypersensitive ABR thresholds. How long that hyper-sensitive state might last and, given our findings that ABR P1 amplitudes seem only to transiently change following noise, what mechanism(s) underlie this heightened state of sensitivity must be further studied. Related to the innate immune cell presence within the cochlea, loss of GR from Sox9^+^-supporting cells resulted in an increase in Iba1^+^ cells observed prior to noise exposure but led to a decreased innate immune response driven by noise assessed at 7 dpn. It will be important to further define the dynamic response over time to ensure that the timeframe over which data were collected did not miss a shift in maximal Iba1^+^ cells present in the cochlea following noise. Sox9^+^-supporting cell MR gene manipulation did not alter either baseline or noise-induced innate immune responses assessed by counting Iba1^+^ cells but transiently influenced ABR P1 neural amplitude.

## 4. Materials and Methods

### 4.1. Production of Compound Transgenic Mouse Lines, Breeding and Genotyping

Double-transgenic mice were generated by breeding mice carrying either lox P sites flanking exon 2 of the Nr3c1 gene encoding the GR (B6.129S6-Nr3c1tm2.1Ljm/J, JAX stock #012914) or lox P sites flanking exons 5–6 of the Nr3c2 gene encoding the MR (maintained on a C57BL/6J background, gift of Dr. Iris Jaffe, Tufts Univ. School of Medicine) with mice carrying an inducible Cre recombinase transgene driven by promoter elements of the Sox9 gene (Tg(Sox9-cre/ERT2)1Msan/J, JAX stock 018829). Mice were genotyped with standard PCR procedures from tail snips collected at weaning age. Briefly, samples were digested 2–18 h in 500 µL tail lysis buffer (100 mM Tris-HCl, pH8.5, 5 mM EDTA, 200 mM NaCl, 0.2% SDS) containing 100 mg/mL Proteinase K (Fisher, Waltham, MA, USA) at 56 °C on an orbital shaker followed by precipitation of genomic DNA in an equal volume of 100% isopropanol. DNA was recovered and redissolved in 500 µL dH_2_O for use in PCR genotyping reactions. PCR primer sequences (all listed as 5′ to 3′ in orientation) were as follows: floxed GR primer set: fGR1 AATCAGAATTGCTCACTCACAA; fGR2 CAGTGTTACTACTTCCAGTTC producing a 290 bp mutant band or a 219 bp WT band; floxed MR primer sets: wild type allele—MR1 CCA CTT GTA TCG GCA ATA CAG TTT AGT GTC, MR2 CAC ATT GCA TGG GGA CAA CTG ACT TC, producing a 214 bp WT band; floxed allele—MR3 CTG TGA TGC GCT CGG AAA CGG, MR4 GGA GAT CGT ACA AAC ATA CGA ACA GC, producing a 565 bp mutant band. Sox9iCre transgene expression was checked by PCR for each line using the following primers: Sox9Cre1 GCG GTG TGG CAG TAA AAA CTA TC, Sox9Cre2 GTG AAA CAG CAT TGC TGT CAC TT, producing a 100 bp Tg band; IntCon1 CTA GGC CAG AGA ATT GAA AGA TCT, IntCon2 GTA GGT GGA AAT TCT AGC ATC ATC C, producing a 324 bp loading control band (present in all lanes when DNA was loaded to ensure that a negative lane was the product of the lack of transgene and not a missed DNA load).

### 4.2. Housing

Mice were housed in a standard ventilated cage rack system in the UMMC vivarium (12 h light/dark cycle, food and water ad libitum, ambient sound) until used in experiments. Mice were screened for absence of middle-ear infection prior to inclusion in experiments (no mice were excluded due to middle-ear infection). Mice of both sexes were used.

### 4.3. Tamoxifen Injections

To generate conditional knockouts (cKO), two- to five-month-old f/f GR:Sox9iCre mice, f/+ GR:Sox9iCre mice, and f/f MR:Sox9iCre mice underwent five once-daily injections of tamoxifen (Sigma, St. Louis, MI, USA, 75 mg/kg, IP) previously dissolved in corn oil (Sigma) by heating for 1–2 h at 45 °C. Dissolved tamoxifen was stored in 1 mL aliquots in amber-colored Eppendorf-style tubes at −20 °C further protected from light by using a cardboard freezer box. Prior to use, tamoxifen aliquots were thawed and inspected for precipitate. If tamoxifen was precipitated, the tube was warmed to 45 °C while also being mixed by inversion on a rotator to redissolve the drug. A similarly aged cohort of each floxed line was injected (IP) with corn oil alone to produce control (non-cKO) lines. Mice rested for a minimum of two days and sometimes up to 5 days following the last tamoxifen injection before proceeding with experiments.

### 4.4. Morphological Verification of Mouse Lines as Useful for Experiments

Targeted ablation of the gene of interest can only occur in cells that express both the floxed gene of interest and the inducible Cre recombinase. We first visually assessed whether Sox9 iCre could be activated by tamoxifen delivered IP and concomitantly assessed which cells would undergo tamoxifen-induced Cre recombination. To perform this experiment, we bred mice harboring the Sox9iCre transgene with a ROSA26 flox-stop-flox tdTomato reporter mouse line (Ai14, B6.Cg-Gt(ROSA)26Sor^tm14(CAG-tdTomato)Hze^/J, JAX stock 7914) to generate double transgenic mice (tdTomato:Sox9iCre). Cochleae were processed and inspected via confocal microscopy.

### 4.5. Verification of Mouse Lines as Useful for Experiments-Isolation and Validation of Total RNA from Cochleae

Next, because we targeted only a subset of cells in the cochlea for ablation of each gene of interest, we also used quantitative real-time PCR (qRT-PCR) techniques to assess the degree to which expression of each gene was knocked down. Tamoxifen or corn oil injections were given IP daily for 5 days. Cochlear total RNA was isolated 7 days after the last tamoxifen injection. Mice underwent cervical dislocation and decapitation. Skin was reflected toward the snout, the bulla was localized and removed, and the cochleae were quickly accessed and pulled from the temporal bone using #5 Dumont forceps. Care was taken to ensure no brain remnants were carried along with the isolated inner ears. Tissue isolation for each pool was completed in approximately 30–45 s per mouse. The cochleae were immediately immersed in ice cold 500 μL Trizol Reagent (Invitrogen/Ambion) in an RNase-free 15 mL tube maintained on ice. Immediately following isolation of cochleae for each condition (i.e., before the next group was processed), the pooled cochleae were homogenized using an Omni handheld homogenizer (Omni International) and Omni disposable 7 mm × 110 mm plastic rotor stator generator tips designed for hard tissue (Omni 30750H). Generator tips were changed between pools of tissue from each group. Care was taken to minimize any heat produced by the homogenization process by maintaining the tube and tissue on ice and using the lowest speed necessary to homogenize the tissue. Homogenization was completed within 5–6 min from the start of tissue isolation for each pool. Homogenized samples were maintained on ice until all group samples were homogenized. After all tissue from all conditions were homogenized, the samples were transferred to RNase-free 1.6 mL Eppendorf-style tubes and centrifuged at 4 °C to sediment debris. Supernatant was then transferred to Direct-Zol RNA mini-prep Plus columns (Zymo Research) and total RNA was isolated following the manufacturer’s instructions. This included an in-column 15 min DNase I treatment (room temperature) to remove genomic DNA from the sample. Total RNA was recovered in 30 μL RNase-free PCR grade water (Ambion). Assessing integrity of samples and sample purity are important starting points for a robust qPCR assay and are critical to understanding PCR reaction efficiency (in addition to proper primer design). Nanodrop assessed purity (A260/A280) ranged from 1.903 to 1.995. RNA yields were analyzed using RNA binding dyes. A Qubit 4 fluorometry Qubit RNA HS Assay kit was used, and results ranged from 19 ng/mL to 432 ng/μL. RNA integrity was assessed with RNA binding dyes using Qubit 4 fluorometry and the Qubit RNA IQ assay kit. All samples had an integrity score above 75%.

#### Verification of Mouse Lines as Useful for Experiments—qRT-PCR Procedures

To establish whether tamoxifen injections in the floxed GR:Sox9iCre and floxed MR:Sox9iCre double transgenic mice produced modifications of the target genes of interest, a SYBR Green-based qRT-PCR approach was taken. cDNA was produced from the total RNA isolated from the cochleae of each group as described above. The Bio-Rad iScript cDNA synthesis kit was used following the manufacturer’s instructions. An amount of 50 ng of cDNA was used per PCR reaction. qRT-PCR was carried out as triplicate technical repeats on the biological duplicate samples. Reactions were run on a Bio-Rad CFX96 Touch Real-Time PCR Detection System (UMMC Genomics core).

Design of the qRT-PCR followed a set of core MIQE guidelines [47,129]. Three primer sets for each gene of interest were designed with the online IDT primer design system and targeted exon 2, the floxed exon, of each gene of interest. Selected sequences were submitted to the NCBI Primer-Blast and compared against the mouse genome to ensure no amplification of spurious product might occur. Prior to performing the qRT-PCR reaction on the cochlear samples, the PCR amplification efficiency of all primer sets was first tested on cDNA generated from whole mouse brain total RNA following the same steps used for cochlear samples. Production of single amplicons for each primer set was established via melt curve analysis at the conclusion of the mouse-brain test PCR run. One primer set for each gene of interest performing best in terms of both amplification efficiency and production of single amplicons evidenced by a single melt curve peak from the test brain sample was then used with cochlear cDNA to quantify tamoxifen-induced effects on expression levels. Triplicates of cDNA from each group were run. Primers used for qRT-PCR were as follows: floxed GR—Nr3c1ex2FWD: 5′ GGA AGC GTG ATG GAC TTG TAT 3′, Nr3c1ex2REV: 5′ GCTTGG AAT CTG CCT GAG AA 3′; floxed MR—Nr3c2ex2FWD: 5′ GAG AGA TGC CGA GTA CAC TTA TG 3′, Nr3c2ex2REV 5′ GGA CCT GTG ACC ATT CTC TTT 3′. Two genes, beta actin and GAPDH, were used as comparators, serving as control genes (unchanging expression level) on which the qRT-PCR ∆∆Ct calculations were based. Use of averaged multiple reference genes guards against unexpected changes to reference genes that can skew ∆∆Ct calculations when only one reference gene is used and is not fully validated as unchanging under the experimental conditions being surveyed [47,130]. Arithmetic and geometric means were virtually identical for control genes (and thus by the arithmetic-geometric mean inequality, suggestive that the expression levels were similar between reference genes), so a simple arithmetic mean of the control genes expression levels was used in calculating potential expression level changes of the genes of interest (note that the arithmetic mean will always be larger than the geometric mean unless the values are identical and therefore the decision to use one or the other mean will slightly alter final differential expression values of genes of interest). Primer sequences for these genes were as follows: beta actin—ACTB-fwd: 5′ GGC TGT ATT CCC CTC CAT C 3′, ACTB-rev: 5′ CCA GTT GGT AAC AAT GCC ATG T 3′, for GAPDH—GAPDH-fwd: 5′ AGG TCG GTG TGA ACG GAT TTG 3′, GAPDH-rev: 5′ TGT AGA CCA TGT AGT TGA GGT CA 3′.

The design for the qRT-PCR was as follows. Two main groups based on treatment were created for each line of mice (fGR or fMR). For the fGR line, there were two groups based on whether tamoxifen was administered (fGR with tamoxifen) or not (fGR, no tamoxifen). The same was done for the fMR line (fMR, with tamoxifen; fMR, no tamoxifen). Thus, the main groups created reflect a genotype x condition grouping. For each condition of each mouse line, two groups of 4 mice each were created. Cochleae from the 4 mice of each group were collected, homogenized together, and total RNA was extracted. This created two replicate pools of cochlear total RNA for each genotype x condition main group. The total RNA samples isolated from the two groups of 4 mice each are biological replicates, having come from the same genotype x condition main group but representing a different pool of mice. Thus, a total of 8 mice were used to produce 2 biological replicate groups (4 mice per biological replicate). The biological replicates (the two groups of pooled cochleae from 4 mice each) were then used to run triplicate technical repeats of the qRT-PCR. Data from the triplicate repeats of each biological repeat were then used for statistical analysis of gene expression level changes. The final data represented by the box and whisker plots of Figure 2 are the statistical assessment of the combined qPCR data gathered over the two biological repeats, each of which is composed of the triplicate technical repeats, normalized to the reference genes. An example of the experimental design is found in Supplementary Materials Figure S4.

Results of all PCR reactions were analyzed with CFX Maestro software. The extended methodological description here and results provided in the Supplementary Materials section are provided to ensure that MIQE guidelines are met with respect to primer design, similar amplification efficiency across reactions (see results) and purity/lack of contamination of starting material. From this, the ∆∆Ct calculation is a viable estimate of tamoxifen-induced effects on the genes of interest.

### 4.6. Auditory Physiology—ABRs and DPOAEs

Mice received 5 daily Tamoxifen injections. Minimally two days (and more often at least 5 days) elapsed between the last tamoxifen injection and collection of basal ABR data. Prior to auditory physiological measurements, mice were housed in a walk-in sound-attenuating chamber (Industrial Acoustics Co., North Aurora, IL, USA,).

For all lines of mice, both sexes were used. To assess sex effects, we used Bayesian inferential modeling. Otherwise, data from both sexes were compiled and analyzed in the same batch. Auditory thresholds, peak 1 amplitudes and latencies were assessed via the auditory brainstem response (ABR). The 2f1–f2 distortion product otoacoustic emission (DPOAE) amplitudes were also recorded. Mice were anaesthetized with a mixture of ketamine (100 mg/kg, IP) and xylazine (10 mg/kg, IP). Body temperature was maintained at 37 ± 1 °C with a heating pad controlled by monitoring body temperature via a rectal probe (FHC temperature regulation system). Subcutaneous needle leads were placed at the tail (ground), under the right pinna (reference) and at the vertex of the head (active). A custom produced speaker/microphone probe (contact Eaton Peabody Labs (Boston, MA, USA) engineering for stereolithography files) was lowered to the outside of the right ear canal. Custom LabView software (Cochlear Function Test Suite, Eaton Peabody Labs (Boston, MA, USA), Mass. Eye and Ear Infirmary) was used to present sound stimuli to the ear canal and record DPOAEs and ABRs. Recording frequencies spanned from 5.66 to 45.25 kHz and were presented in half-octave steps as previously described [112]. The ABR neural trace stack and the 2f1–f2 DPOAE was saved to the lab computer and then exported to a personal laptop for offline analysis. The ABR operator recorded initial decisions of ABR thresholds. As a secondary check on threshold, ABR Peak Analysis software was used to independently identify auditory thresholds and determine peak amplitude and latency of ABR wave 1 [131] (for software code see [132]).

#### Auditory Physiology—Noise Exposure

Those mice receiving noise exposure underwent the tamoxifen injections and basal ABR data gathering as described above. A minimum of 2 days (and up to 5 days) after basal ABR data gathering, mice were exposed to noise. Thus, 9 to as many as 15 days elapsed between the first tamoxifen injections and noise exposure. Custom LabView software (Eaton Peabody Labs, MEEI) was used to generate octave-band noise (8–16 kHz). A maximum of two mice were placed in a wire mesh cage (6 × 6 × 6 in), and up to four cages were placed within a custom-built noise exposure chamber on a rotating platform. Awake mice were exposed to noise for 2 h, beginning at approximately 10:00 a.m. (to control circadian rhythm influences on physiology). Mice were visually monitored over the entire span of the noise exposure using a standard wireless baby monitor system composed of camera and associated screen. Noise intensity was constantly measured from a centrally located free-field microphone (PCB Piezotronics) hung approximately to the level of the head of a mouse. Prior inspection of the noise exposure chamber revealed that noise intensity varied within ± 0.5 dB SPL around the target intensity and across the space occupied by the mouse cages within the exposure box. As prior studies have demonstrated a role of GR after traumatic noise exposure, our experiments with fGR:Sox9iCre double-transgenic mice used 94 dB SPL to assess vulnerability to non-neuropathic noise [133]. With fewer studies investigating the role of MR after noise exposure [13], fMR:Sox9iCre double-transgenic mice were exposed to 100 dB SPL intensity noise with the expected outcome to drive noise-induced injury in these mice.

### 4.7. Neuroanatomical Analyses-Tissue Preparation and Immunofluorescent Labeling

Mice were transcardially perfused first with 1× PBS (vascular rinse), followed by 10 mL room temperature 0.1 M sodium phosphate buffered 4% paraformaldehyde (PFA). Immediately following perfusion of fixative, a lateral approach was used to access the cochleae. Cochleae were slowly perfused via the round and oval windows with fixative (approximately 1 mL), dissected free of the skull, and postfixed in the same fixative for between 30 minutes and 2 hours dependent on requirements. The cochleae were then decalcified in 8% EDTA in 1× PBS overnight on a rotator either at 4 °C or room temperature. For wholemount sensory epithelium preparations used for afferent synapse and Iba1^+^ immune cell visualization, cochleae were dissected into apical, middle and basal pieces by removing the lateral wall and tectorial membrane and cutting into regions with spring scissors [134,135].

Tissue used for immunolabeling afferent synapses was cryoprotected in 30% sucrose for approximately 20–30 min, permeabilized by freeze/thawing at −80 °C, rinsed three times in 1× PBS, and then blocked in 5% normal donkey serum (Jackson ImmunoResearch) with 0.5% Triton X−100 (Sigma, St. Louis, MI, USA) in PBS for 1 hour at room temp. For immunostaining of synaptic profiles, rabbit monoclonal anti-CtBP2 (Abcam, 1:200, Cambridge, UK), and mouse anti-GluA2 (Millipore, 1:1000, Billerica, MA, USA) primary antibodies were diluted in 1× PBS with 1% normal donkey serum and 0.1% Triton X−100. Tissue was incubated in the primary antibody cocktail overnight at 37 °C. The following morning, tissue was rinsed 3 times in 1× PBS, then incubated 2 hours at 37 °C with Alexa labeled secondary antibodies (Alexa 488 anti-mouse IgG2A and Alexa 594 anti-rabbit, both 1:1000 dilution) with 0.1% Triton X−100 and 1% normal serum. Tissue was incubated at room temperature for 2 h. Following three 5 minutes rinse in 1× PBS, tissue underwent final trimming to ensure the specimens were flat and then coverslipped with SlowFade Gold with DAPI (Invitrogen, Carlsbad CA, USA).

For immunofluorescence labeling of immune cells, cochlear whole-mount pieces were blocked 1 hour at room temperature in 5% normal donkey serum, incubated overnight at 4 °C with rabbit monoclonal anti-Iba1 (1:1000) primary antibody (Abcam), washed three times for 5 minutes each in 1× PBS, then incubated 2 hours at 37 °C with Alexa 594 anti-rabbit secondary antibody. Wholemount cochleae were mounted on microscope slides with antifade FluoroGold with DAPI (Pierce/Fisher, Waltham, MA, USA) and coverslipped.

To visualize Sox9-driven Cre-mediated recombination, SoxiCre:tdTomato double transgenic mice were administered tamoxifen as described above. Mice were perfused with aldehyde fixative and cochleae isolated, as described above. Cochleae were decalcified in 8% EDTA for three days at 4 °C. The cochlear apex was opened, and cochleae were cryopreserved through sequential graded sucrose incubations (10%, 15%) before OCT embedding. Cryostat sections were cut at 12 microns and placed onto gelatin double subbed slides. Sections were incubated overnight at room temperature with anti-RFP (Rockland, 1:1000) primary antibody in 1% normal donkey serum with 0.1% Triton X−100. Incubation proceeded in a humidified chamber. Slides were then washed with 1× PBS, incubated for 1 h at room temperature with Alexa 594 anti-rabbit secondary antibody in the same diluent as the primary incubation. Following final 1× PBS washes, cochleae were DAPI counterstained and mounted and coverslipped with Fluorogel (Electron Microscopy Sciences, Darmstadt, Germany).

#### Neuroanatomical Analyses-Confocal Imaging and Image Analysis

Immunohistochemically stained tissue was imaged on a Zeiss LSM 880 confocal microscope. A custom ImageJ2 plugin (Eaton Peabody Labs, Boston, MA, USA, (MEEI)) was used to determine approximate cochlear-place frequency in mouse cochlear wholemounts [136]. Images of immunostained cochlear pieces from the 22.6 kHz region were obtained using a 40×/1.3 NA oil immersion objective and 3.5× digital zoom. Layers through the z-stack were scanned at one-micron intervals with a resolution of 0.06 μm per pixel. For immune cells in the sensory epithelium, the middle turn was imaged with a 20×/0.8 NA objective and 0.6× digital zoom. Layers through the z-stack were scanned at two-micron intervals with a resolution of 0.69 μm per pixel.

Images were exported to ImageJ (1.53o) for analysis [137]. Inner hair cell nuclei were marked using the Cell Counter plugin. The puncta channels were passed through a filter with 0.7 μm rolling ball radius. From maximal intensity projections, an ImageJ2 plugin was used as a first pass puncta detection with default settings to determine mean puncta intensities [138]. The images were then median filtered with 0.3 μm diameter to develop the background intensity. The channel background was subtracted from each slice and tiffs were generated for synapse detection using SynapseJ (v.1) [139]. Detection threshold was set to one-fourth the mean puncta intensity per channel. A median blur of 0.3 μm was applied and puncta size had a maximum diameter of 1.4 μm. Default noise and Find Maxima were applied. Detected puncta were imported into R and processed using the following packages: readr (2.1.2), rlang (1.0.6), tidyverse (1.3.2), reshape2 (1.4.4), rgdal (1.5.32), spatstat (2.3.4), and nabor (0.5.0). Orphaned ribbons and synapses were observer classified from raster plots of 1.5 × 1.5 × 3 μm (X × Y × Z) voxel space around detected CtBP2 puncta. Counts were divided by the number of IHC in the image (typically 5–7 IHCs) to arrive at an average count per hair cell. Three or four images taken of the 22.6 kHz region of each cochlea. Middle turn segments were brightness and contrast adjusted for counting Iba1^+^-cells with the Cell Counter ImageJ plugin. The sensory epithelium was observer delimited by tracing a line through the IHC nuclei and then though the outer sulcus cells using DAPI stain as a guide. The shape of the sensory epithelium was reconstructed using the sf package (1.0.8). Immune cells in the vicinity of the sensory epithelium counted and their morphology was further analyzed. From summed intensity projections, the cell soma was outlined using the polygon selection tool and branches were traced using the segmented lines tool.

### 4.8. Data Analysis

Analysis was conducted in R (version 4.2.1 (2022–06−23 ucrt)) using the following packages: lme4 (1.1.30), lmerTest (3.1.3), afex (1.1.1), emmeans (1.8.0). Numbers of mice for each analysis are indicated in figure legends or the results section. Physiological data collection was designed for within-group recovery analysis. The statistical approach emphasized comparison to baseline values with Dunnett-equivalent correction while pairwise comparisons were performed with Tukey correction. Linear mixed-effects models were chosen to consider recovery dynamics of growth-curve data. Additionally, analysis of ABR P1 amplitudes and ABR P1 latencies were limited to a sound stimulus of 50 or 60 dB SPL, respectively. Mixed effects for the best model specified day as a nested effect within each mouse and slopes which varied by sound-intensity level. The full mixed effects were not supported for all datasets in which case slopes were not allowed to vary by level for that model. To analyze data collected over multiple images per mouse, image was treated as a nested effect within mouse. Denominator degrees of freedom and F-statistics were computed with the Satterthwaite method. Data were plotted as mean ± SEM. Significance was set at *p* < 0.05.

A Bayesian approach was chosen for analysis of sex effects following the rationale of Saber et al. [52]. Mixed-effects models have suppressed type I error rates but inflated type II error rates with small sample sizes and increasing model variables. Bayesian models can handle small sample sizes and produce reliable effect estimates with weakly informative priors on the expected data distribution. Bayesian inference further supports reproducibility of results from variable or mild injury types, such as mild noise exposure, enabling discussion of probability and magnitude of injury effects. To model data in a Bayesian inferential framework, we used the Stan computational platform (rstan, 2.26.13) implemented with the brms package (2.17.0). Weakly informative priors were placed on model parameters and variance components [140]. ABR P1 amplitude data with 80 dB sound stimulus were fit to a gaussian regression model. Normal priors, ~Normal(0, 1), were set to population-level parameters (e.g., male [coded for Sex]) and the intercept to constrain posterior draws to expected sample space. A half-Cauchy prior, ~Cauchy(0, 5), was placed on the residual variance as recommended for hierarchical models. Random intercepts varied by individual mice and group-level variance was estimated by genotype. The model was fit with four Markov chains: burn-in was 2000 iterations followed by 3000 sampling iterations, culminating in 12,000 posterior iterations per effect estimate. Convergence of estimates was numerically assessed with potential scale reduction factor ($\hat{R}$) and effective sample sizes ($n_{eff}$) with optimal values of $\hat{R}$ = 1.0–1.1 and $n_{eff}$ > 1000. Convergence and sampling behavior was viewed with trace plots and auto-correlation plots. Posterior sampling was visually assessed with bayesplot (1.9.0) to produce posterior predictive checks with 1000 draws over the actual data. Stability of estimates was assessed by doubling iterations and calculating effect bias. Effect estimates were plotted as median values with quantile interval bars depicting 66% and 95% credible intervals (CrI) using tidybayes (3.0.2). To aid interpretation, we calculated a point-based Bayes Factor (BF_10_) for the null-hypothesis density ratio with the Savage–Dickey method [141,142]. For instance, BF_10_ = 2 supports the model such that the actual data are twice less likely to contain a compared data distribution of point value after seeing the data. A BF_10_ < 1 indicates support for the null hypothesis whereas a BF_10_ > 3 and <10 provides moderate support that two distributions are distinct or do not contain a value of interest (i.e., a value of 0 for baseline normalized distributions) [143]. While we have calculated BF_10_ for some estimations, we primarily consider estimates in terms of credible intervals which in the results are represented graphically (median + CrI).

## Data Availability

Data is available within the article and Supplementary Materials. A GitHub repository for custom R functions used to process some of the data presented in the manuscript can be accessed at: https://github.com/ccaiman/data_processing.

## Supplementary Materials

The following supporting information can be downloaded at https://www.mdpi.com/article/10.3390/ijms24043320/s1.

## References

[1] Sturm J.J., Maurrasse S.E., Golub J.S. (2021). Intrantympanic steroid injection. Oper. Tech. Otolaryngol. Head Neck Surg..

[2] Ajduk J., Košec A., Kelava I., Ries M., Gregurić T., Kalogjera L. (2019). Recovery from Sudden Sensorineural Hearing Loss May Be Linked to Chronic Stress Levels and Steroid Treatment Resistance. Am. J. Audiol..

[3] Kil S.-H., Kalinec F. (2013). Expression and dexamethasone-induced nuclear translocation of glucocorticoid and mineralocorticoid receptors in guinea pig cochlear cells. Hear. Res..

[4] Ong G.S.Y., Young M.J. (2017). Mineralocorticoid regulation of cell function: The role of rapid signalling and gene transcription pathways. J. Mol. Endocrinol..

[5] Jin D.X., Lin Z., Lei D., Bao J. (2009). The role of glucocorticoids for spiral ganglion neuron survival. Brain Res..

[6] De Kloet E.R., de Kloet S.F., de Kloet C.S., de Kloet A.D. (2019). Top-down and bottom-up control of stress-coping. J. Neuroendocr..

[7] Tahera Y., Meltser I., Johansson P., Bian Z., Stierna P., Hansson A., Canlon B. (2006). NF-kappaB mediated glucocorticoid response in the inner ear after acoustic trauma. J. Neurosci. Res..

[8] Tahera Y., Meltser I., Johansson P., Hansson A.C., Canlon B. (2006). Glucocorticoid receptor and nuclear factor-kappa B interactions in restraint stress-mediated protection against acoustic trauma. Endocrinology.

[9] Lee S.-H., Lyu A.-R., Shin S.-A., Jeong S.-H., Lee S.-A., Park M.J., Park Y.-H. (2019). Cochlear Glucocorticoid Receptor and Serum Corticosterone Expression in a Rodent Model of Noise-induced Hearing Loss: Comparison of Timing of Dexamethasone Administration. Sci. Rep..

[10] MacArthur C., Hausman F., Kempton B., Trune D.R. (2015). Intratympanic Steroid Treatments May Improve Hearing via Ion Homeostasis Alterations and Not Immune Suppression. Otol. Neurotol..

[11] Halonen J., Hinton A.S., Frisina R.D., Ding B., Zhu X., Walton J.P. (2016). Long-term treatment with aldosterone slows the progression of age-related hearing loss. Hear. Res..

[12] Nelson L., Lovett B., Johns J.D., Gu S., Choi D., Trune D., Hoa M. (2021). In silico Single-Cell Analysis of Steroid-Responsive Gene Targets in the Mammalian Cochlea. Front. Neurol..

[13] Singer W., Kasini K., Manthey M., Eckert P., Armbruster P., Vogt M.A., Jaumann M., Dotta M., Yamahara K., Harasztosi C. (2018). The glucocorticoid antagonist mifepristone attenuates sound-induced long-term deficits in auditory nerve response and central auditory processing in female rats. FASEB J..

[14] Marchetta P., Eckert P., Lukowski R., Ruth P., Singer W., Rüttiger L., Knipper M. (2022). Loss of central mineralocorticoid or glucocorticoid receptors impacts auditory nerve processing in the cochlea. Iscience.

[15] Hirose K., Discolo C.M., Keasler J.R., Ransohoff R. (2005). Mononuclear phagocytes migrate into the murine cochlea after acoustic trauma. J. Comp. Neurol..

[16] Tornabene S.V., Sato K., Pham L., Billings P., Keithley E.M. (2006). Immune cell recruitment following acoustic trauma. Hear. Res..

[17] Okano T., Nakagawa T., Kita T., Kada S., Yoshimoto M., Nakahata T., Ito J. (2008). Bone marrow-derived cells expressing Iba1 are constitutively present as resident tissue macrophages in the mouse cochlea. J. Neurosci. Res..

[18] Frye M.D., Zhang C., Hu B.H. (2018). Lower level noise exposure that produces only TTS modulates the immune homeostasis of cochlear macrophages. J. Neuroimmunol..

[19] Kaur T., Clayman A.C., Nash A.J., Schrader A.D., Warchol M.E., Ohlemiller K.K. (2019). Lack of Fractalkine Receptor on Macrophages Impairs Spontaneous Recovery of Ribbon Synapses after Moderate Noise Trauma in C57BL/6 Mice. Front. Neurosci..

[20] Cederroth C.R., Park J.-S., Basinou V., Weger B.D., Tserga E., Sarlus H., Magnusson A.K., Kadri N., Gachon F., Canlon B. (2019). Circadian Regulation of Cochlear Sensitivity to Noise by Circulating Glucocorticoids. Curr. Biol..

[21] Yang S., Cai Q., Vethanayagam R.R., Wang J., Yang W., Hu B.H. (2016). Immune defense is the primary function associated with the differentially expressed genes in the cochlea following acoustic trauma. Hear. Res..

[22] Cai Q., Vethanayagam R.R., Yang S., Bard J., Jamison J., Cartwright D., Dong Y., Hu B.H. (2014). Molecular profile of cochlear immunity in the resident cells of the organ of Corti. J. Neuroinflamm..

[23] Monzack E.L., Cunningham L.L. (2013). Lead roles for supporting actors: Critical functions of inner ear supporting cells. Hear. Res..

[24] Ramírez-Camacho R., García-Berrocal J.R., Trinidad A., González-García J.A., Verdaguer J.M., Ibáñez A., Rodríguez A., Sanz R. (2006). Central role of supporting cells in cochlear homeostasis and pathology. Med. Hypotheses.

[25] Vetter D.E., Yee K.T., Ramkumar V., Rybak L.P. (2018). Corticotropin-releasing factor signaling in the mammalian cochlea: An integrative niche for cochlear homeostatic balance against noise. Inflammatory Mechanisms in Mediating Hearing Loss.

[26] Anttonen T., Herranen A., Virkkala J., Kirjavainen A., Elomaa P., Laos M., Liang X., Ylikoski J., Behrens A., Pirvola U. (2016). c-Jun N-Terminal Phosphorylation: Biomarker for Cellular Stress Rather than Cell Death in the Injured Cochlea. Eneuro.

[27] Herranen A., Ikäheimo K., Virkkala J., Pirvola U. (2018). The Stress Response in the Non-sensory Cells of the Cochlea under Pathological Conditions—Possible Role in Mediating Noise Vulnerability. J. Assoc. Res. Otolaryngol..

[28] Kalinec F., Webster P., Maricle A., Guerrero D., Chakravarti D.N., Chakravarti B., Gellibolian R., Kalinec G. (2009). Glucocorticoid-stimulated, transcription-independent release of annexin A1 by cochlear Hensen cells. Br. J. Pharmacol..

[29] Hayashi Y., Suzuki H., Nakajima W., Uehara I., Tanimura A., Himeda T., Koike S., Katsuno T., Kitajiri S.-I., Koyanagi N. (2020). Cochlear supporting cells function as macrophage-like cells and protect audiosensory receptor hair cells from pathogens. Sci. Rep..

[30] Frieler R.A., Meng H., Duan S.Z., Berger S., Schütz G., He Y., Xi G., Wang M.M., Mortensen R.M. (2011). Myeloid-Specific Deletion of the Mineralocorticoid Receptor Reduces Infarct Volume and Alters Inflammation during Cerebral Ischemia. Stroke.

[31] Chantong B., Kratschmar D.V., Nashev L.G., Balazs Z., Odermatt A. (2012). Mineralocorticoid and glucocorticoid receptors differentially regulate NF-kappaB activity and pro-inflammatory cytokine production in murine BV-2 microglial cells. J. Neuroinflamm..

[32] Trune D.R., Kempton J.B., Gross N.D. (2006). Mineralocorticoid receptor mediates glucocorticoid treatment effects in the autoimmune mouse ear. Hear. Res..

[33] Creber N.J., Eastwood H.T., Hampson A.J., Lo J., Zhang D., Chambers S.A., Bester C.W., Thorne P.R., O’Leary S.J. (2022). Spironolactone Ameliorates Cochlear Implant Induced Endolymphatic Hydrops. Otol. Neurotol..

[34] Degerman E., In’t Zandt R., Palbrink A., Magnusson M. (2019). Endolymphatic hydrops induced by different mechanisms responds differentially to spironolactone: A rationale for understanding the diversity of treatment responses in hydropic inner ear disease. Acta Oto-Laryngol..

[35] Vetter D.E., Li C., Zhao L., Contarino A., Liberman M.C., Smith G.W., Marchuk Y., Koob G.F., Heinemann S.F., Vale W. (2002). Urocortin-deficient mice show hearing impairment and increased anxiety-like behavior. Nat. Genet..

[36] Zhang Y., Zhang S., Zhang Z., Dong Y., Ma X., Qiang R., Chen Y., Gao X., Zhao C., Chen F. (2020). Knockdown of Foxg1 in Sox9+ supporting cells increases the trans-differentiation of supporting cells into hair cells in the neonatal mouse utricle. Aging Albany NY.

[37] Stone J.S., Wisner S.R., Bucks S.A., Lagarde M.M.M., Cox B.C. (2018). Characterization of Adult Vestibular Organs in 11 CreER Mouse Lines. J. Assoc. Res. Otolaryngol..

[38] Wonkam A., Adadey S.M., Schrauwen I., Aboagye E.T., Wonkam-Tingang E., Esoh K., Popel K., Manyisa N., Jonas M., Dekock C. (2022). Exome sequencing of families from Ghana reveals known and candidate hearing impairment genes. Commun. Biol..

[39] Scheffer D.I., Shen J., Corey D.P., Chen Z.-Y. (2015). Gene Expression by Mouse Inner Ear Hair Cells during Development. J. Neurosci..

[40] Shen J., Scheffer D.I., Kwan K.Y., Corey D.P. (2015). SHIELD: An integrative gene expression database for inner ear research. Database Oxford.

[41] Mak A.C., Szeto I.Y., Fritzsch B., Cheah K.S. (2009). Differential and overlapping expression pattern of SOX2 and SOX9 in inner ear development. Gene Expr. Patterns.

[42] Yao X., Rarey K.E. (1996). Localization of the Mineralocorticoid Receptor in Rat Cochlear Tissue. Acta Oto-Laryngol..

[43] Furuta H., Mori N., Sato C., Hoshikawa H., Sakai S.-I., Iwakura S., Doi K. (1994). Mineralocorticoid type I receptor in the rat cochlea: mRNA identification by polymerase chain reaction (PCR) and in situ hybridization. Hear. Res..

[44] Sinha P.K., Pitovski D.Z. (1995). [^3^H]-Aldosterone Binding Sites (Type I Receptors) in the Lateral Wall of the Cochlea: Distribution Assessment by Quantitative Autoradiography. Acta Oto-Laryngol..

[45] Pitovski D.Z., Drescher M.J., Drescher D.G. (1993). High affinity aldosterone binding sites (Type I receptors) in the mammalian inner ear. Hear. Res..

[46] Pitovski D.Z., Drescher M.J., Drescher D.G. (1994). Glucocorticoid receptors in the mammalian inner ear: RU 28362 binding sites. Hear. Res..

[47] Bustin S.A., Benes V., Garson J.A., Hellemans J., Huggett J., Kubista M., Mueller R., Nolan T., Pfaffl M.W., Shipley G.L. (2009). The MIQE Guidelines: Minimum Information for Publication of Quantitative Real-Time PCR Experiments. Clin. Chem..

[48] Ruiz-Villalba A., Ruijter J.M., van den Hoff M.J.B. (2021). Use and Misuse of C(q) in qPCR Data Analysis and Reporting. Life.

[49] Cody A.R., Johnstone B.M. (1981). Acoustic trauma: Single neuron basis for the “half-octave shift”. J. Acoust. Soc. Am..

[50] Budak M., Grosh K., Sasmal A., Corfas G., Zochowski M., Booth V. (2021). Contrasting mechanisms for hidden hearing loss: Synaptopathy vs myelin defects. PLoS Comput. Biol..

[51] Ramamoorthy S., Nuttall A.L. (2012). Half-Octave Shift in Mammalian Hearing Is an Epiphenomenon of the Cochlear Amplifier. PLoS ONE.

[52] Saber M., Giordano K.R., Hur Y., Ortiz J.B., Morrison H., Godbout J.P., Murphy S.M., Lifshitz J., Rowe R.K. (2020). Acute peripheral inflammation and post-traumatic sleep differ between sexes after experimental diffuse brain injury. Eur. J. Neurosci..

[53] Fernandez K.A., Guo D., Micucci S., De Gruttola V., Liberman M.C., Kujawa S.G. (2020). Noise-induced Cochlear Synaptopathy with and without Sensory Cell Loss. Neuroscience.

[54] Shuster B., Casserly R., Lipford E., Olszewski R., Milon B., Viechweg S., Davidson K., Enoch J., McMurray M., Rutherford M.A. (2021). Estradiol Protects against Noise-Induced Hearing Loss and Modulates Auditory Physiology in Female Mice. Int. J. Mol. Sci..

[55] Milon B., Mitra S., Song Y., Margulies Z., Casserly R., Drake V., Mong J.A., Depireux D.A., Hertzano R. (2018). The impact of biological sex on the response to noise and otoprotective therapies against acoustic injury in mice. Biol. Sex Differ..

[56] Martinez-Monedero R., Liu C., Weisz C., Vyas P., Fuchs P.A., Glowatzki E. (2016). GluA2-Containing AMPA Receptors Distinguish Ribbon-Associated from Ribbonless Afferent Contacts on Rat Cochlear Hair Cells. Eneuro.

[57] Mitchell C., Brummett R., Vernon J. (1977). Frequency Effects of Temporary N1 Depression Following Acoustic Overload. Arch. Otolaryngol. Neck Surg..

[58] Davis H., Morgan C.T., Hawkins J.E., Galambos R., Smith F.W. (1950). Temporary deafness following exposure to loud tones and noise. Acta Oto-Laryngol. Suppl..

[59] Cody A.R., Johnstone B.M. (1980). Single auditory neuron response during acute acoustic trauma. Hear. Res..

[60] Chen Z., Peppi M., Kujawa S.G., Sewell W.F. (2009). Regulated Expression of Surface AMPA Receptors Reduces Excitotoxicity in Auditory Neurons. J. Neurophysiol..

[61] Liberman L.D., Suzuki J., Liberman M.C. (2015). Dynamics of cochlear synaptopathy after acoustic overexposure. J. Assoc. Res. Otolaryngol..

[62] Hinwood M., Tynan R.J., Charnley J.L., Beynon S.B., Day T.A., Walker F.R. (2013). Chronic Stress Induced Remodeling of the Prefrontal Cortex: Structural Re-Organization of Microglia and the Inhibitory Effect of Minocycline. Cereb. Cortex.

[63] Kishimoto I., Okano T., Nishimura K., Motohashi T., Omori K. (2019). Early Development of Resident Macrophages in the Mouse Cochlea Depends on Yolk Sac Hematopoiesis. Front. Neurol..

[64] Shin S.H., Jung J., Park H.R., Sim N.S., Choi J.Y., Bae S.H. (2022). The Time Course of Monocytes Infiltration after Acoustic Overstimulation. Front. Cell. Neurosci..

[65] Shin S.H., Yoo J.E., Jung J., Choi J.Y., Bae S.H. (2022). Inflammatory Monocytes Infiltrate the Spiral Ligament and Migrate to the Basilar Membrane after Noise Exposure. Clin. Exp. Otorhinolaryngol..

[66] Rai V., Wood M.B., Feng H., Schabla N.M., Tu S., Zuo J. (2020). The immune response after noise damage in the cochlea is characterized by a heterogeneous mix of adaptive and innate immune cells. Sci. Rep..

[67] Yang W., Vethanayagam R.R., Dong Y., Cai Q., Hu B.H. (2015). Activation of the antigen presentation function of mononuclear phagocyte populations associated with the basilar membrane of the cochlea after acoustic overstimulation. Neuroscience.

[68] Frye M.D., Ryan A.F., Kurabi A. (2019). Inflammation associated with noise-induced hearing loss. J. Acoust. Soc. Am..

[69] Augusto-Oliveira M., Arrifano G.P., Delage C.I., Tremblay M.E., Crespo-Lopez M.E., Verkhratsky A. (2022). Plasticity of microglia. Biol. Rev. Camb. Philos. Soc..

[70] Fernandez-Arjona M.D.M., Grondona J.M., Fernandez-Llebrez P., Lopez-Avalos M.D. (2019). Microglial Morphometric Parameters Correlate with the Expression Level of IL-1beta, and Allow Identifying Different Activated Morphotypes. Front. Cell. Neurosci..

[71] Sakamoto T., Yoshiki M., Sakamoto H. (2017). The mineralocorticoid receptor knockout in medaka is further validated by glucocorticoid receptor compensation. Sci. Data.

[72] Cohen L.D., Ziv N.E. (2019). Neuronal and synaptic protein lifetimes. Curr. Opin. Neurobiol..

[73] Cohen L.D., Zuchman R., Sorokina O., Müller A., Dieterich D.C., Armstrong J.D., Ziv T., Ziv N.E. (2013). Metabolic Turnover of Synaptic Proteins: Kinetics, Interdependencies and Implications for Synaptic Maintenance. PLoS ONE.

[74] Wallace A.D., Cidlowski J.A. (2001). Proteasome-mediated Glucocorticoid Receptor Degradation Restricts Transcriptional Signaling by Glucocorticoids. J. Biol. Chem..

[75] Cate W.-J.F.T., Curtis L.M., Small G.M., Rarey K.E. (1993). Localization of Glucocorticoid Receptors and Glucocorticoid Receptor mRNAs in the Rat Cochlea. Laryngoscope.

[76] Cate W.J.T., Curtis L.M., Rarey K.E. (1992). Immunochemical detection of glucocorticoid receptors within rat cochlear and vestibular tissues. Hear. Res..

[77] Reul J.M., de Kloet E.R. (1985). Two receptor systems for corticosterone in rat brain: Microdistribution and differential occupation. Endocrinology.

[78] Reul J.M., Gesing A., Droste S., Stec I.S., Weber A., Bachmann C., Bilang-Bleuel A., Holsboer F., Linthorst A.C. (2000). The brain mineralocorticoid receptor: Greedy for ligand, mysterious in function. Eur. J. Pharmacol..

[79] Hartmann J., Bajaj T., Klengel C., Chatzinakos C., Ebert T., Dedic N., McCullough K.M., Lardenoije R., Joëls M., Meijer O.C. (2021). Mineralocorticoid receptors dampen glucocorticoid receptor sensitivity to stress via regulation of FKBP. Cell Rep..

[80] Häusl A.S., Brix L.M., Hartmann J., Pöhlmann M.L., Lopez J.-P., Menegaz D., Brivio E., Engelhardt C., Roeh S., Bajaj T. (2021). The co-chaperone Fkbp5 shapes the acute stress response in the paraventricular nucleus of the hypothalamus of male mice. Mol. Psychiatry.

[81] Ding B., Frisina R.D., Zhu X., Sakai Y., Sokolowski B., Walton J.P. (2014). Direct control of Na(+)-K(+)-2Cl(−)-cotransport protein (NKCC1) expression with aldosterone. Am. J. Physiol. Cell Physiol..

[82] Bazard P., Ding B., Chittam H.K., Zhu X., Parks T.A., Taylor-Clark T.E., Bhethanabotla V.R., Frisina R.D., Walton J.P. (2020). Aldosterone up-regulates voltage-gated potassium currents and NKCC1 protein membrane fractions. Sci. Rep..

[83] Hirose K., Liberman M.C. (2003). Lateral Wall Histopathology and Endocochlear Potential in the Noise-Damaged Mouse Cochlea. J. Assoc. Res. Otolaryngol..

[84] Ma Y.L., Gerhardt K.J., Curtis L.M., Rybak L.P., Whitworth C., Rarey K.E. (1995). Combined effects of adrenalectomy and noise exposure on compound action potentials, endocochlear potentials and endolymphatic potassium concentrations. Hear. Res..

[85] Badash I., Quiñones P.M., Oghalai K.J., Wang J., Lui C.G., Macias-Escriva F., Applegate B.E., Oghalai J.S. (2021). Endolymphatic Hydrops is a Marker of Synaptopathy Following Traumatic Noise Exposure. Front. Cell Dev. Biol..

[86] Sewell W.F. (1984). The relation between the endocochlear potential and spontaneous activity in auditory nerve fibres of the cat. J. Physiol..

[87] Wangemann P. (2006). Supporting sensory transduction: Cochlear fluid homeostasis and the endocochlear potential. J. Physiol..

[88] Suzuki S., Ohkusa T., Sato T., Yoshida M., Yasui K., Miwa K., Lee J.-K., Yano M., Kodama I., Matsuzaki M. (2009). Effects of Aldosterone on Cx43 Gap Junction Expression in Neonatal Rat Cultured Cardiomyocytes. Circ. J..

[89] Zhang J., Wang X., Hou Z., Neng L., Cai J., Zhang Y., Shi X. (2020). Suppression of Connexin 43 Leads to Strial Vascular Hyper-Permeability, Decrease in Endocochlear Potential, and Mild Hearing Loss. Front. Physiol..

[90] Wu P.Z., Liberman L.D., Bennett K., de Gruttola V., O’Malley J.T., Liberman M.C. (2019). Primary Neural Degeneration in the Human Cochlea: Evidence for Hidden Hearing Loss in the Aging Ear. Neuroscience.

[91] Heeringa A.N., Köppl C. (2019). The aging cochlea: Towards unraveling the functional contributions of strial dysfunction and synaptopathy. Hear. Res..

[92] Jeng J.Y., Ceriani F., Olt J., Brown S.D.M., Holley M.C., Bowl M.R., Johnson S.L., Marcotti W. (2020). Pathophysiological changes in inner hair cell ribbon synapses in the ageing mammalian cochlea. J. Physiol..

[93] Steenken F., Heeringa A.N., Beutelmann R., Zhang L., Bovee S., Klump G.M., Köppl C. (2021). Age-related decline in cochlear ribbon synapses and its relation to different metrics of auditory-nerve activity. Neurobiol. Aging.

[94] Zhao H.-B., Zhu Y., Liu L.-M. (2021). Excess extracellular K+ causes inner hair cell ribbon synapse degeneration. Commun. Biol..

[95] Katsumi S., Şahin M.I., Lewis R.M., Iyer J.S., Landegger L.D., Stankovic K.M. (2019). Intracochlear Perfusion of Tumor Necrosis Factor-Alpha Induces Sensorineural Hearing Loss and Synaptic Degeneration in Guinea Pigs. Front. Neurol..

[96] Ohlemiller K.K., Jones S.M., Johnson K.R. (2016). Application of Mouse Models to Research in Hearing and Balance. J. Assoc. Res. Otolaryngol..

[97] Cimerman J., Waldhaus J., Harasztosi C., Duncker S.V., Dettling J., Heidrych P., Bress A., Gampe-Braig C., Frank G., Gummer A.W. (2013). Generation of somatic electromechanical force by outer hair cells may be influenced by prestin–CASK interaction at the basal junction with the Deiter’s cell. Histochem. Cell Biol..

[98] Arslan H.H., Satar B., Serdar M., Yilmaz E., Yılmaz E. (2017). Changes in Proinflammatory Cytokines in the Cochlea in Relation to Hearing Thresholds in Noise-Exposed Rats. J. Int. Adv. Otol..

[99] Yang C.-H., Hwang C.-F., Chuang J.-H., Lian W.-S., Wang F.-S., Huang E.I., Yang M.-Y. (2020). Constant Light Dysregulates Cochlear Circadian Clock and Exacerbates Noise-Induced Hearing Loss. Int. J. Mol. Sci..

[100] Li S., Zheng H., Xing Z., Liu Y., Han L., Wang Z., Yu L. (2022). The circadian timing of noise exposure influences noise-induced inflammatory responses in the mouse cochlea. Braz. J. Otorhinolaryngol..

[101] Gibbs J., Ince L., Matthews L., Mei J., Bell T., Yang N., Saer B., Begley N., Poolman T., Pariollaud M. (2014). An epithelial circadian clock controls pulmonary inflammation and glucocorticoid action. Nat. Med..

[102] Lin N., Urata S., Cook R., Makishima T. (2022). Sex differences in the auditory functions of rodents. Hear. Res..

[103] Rouse S.L., Matthews I.R., Li J., Sherr E.H., Chan D.K. (2020). Integrated stress response inhibition provides sex-dependent protection against noise-induced cochlear synaptopathy. Sci. Rep..

[104] Delhez A., Lefebvre P., Péqueux C., Malgrange B., Delacroix L. (2020). Auditory function and dysfunction: Estrogen makes a difference. Cell Mol. Life Sci..

[105] Li Q., Chen M., Zhang C., Lu T., Min S., Li S. (2021). Correction to: Opposite Roles of NT-3 and BDNF in Synaptic Remodeling of the Inner Ear Induced by Electrical Stimulation. Cell. Mol. Neurobiol..

[106] Zuccotti A., Kuhn S., Johnson S.L., Franz C., Singer W., Hecker D., Geisler H.-S., Köpschall I., Rohbock K., Gutsche K. (2012). Lack of Brain-Derived Neurotrophic Factor Hampers Inner Hair Cell Synapse Physiology, but Protects against Noise-Induced Hearing Loss. J. Neurosci..

[107] Parker A., Parham K., Skoe E. (2022). Noise exposure levels predict blood levels of the inner ear protein prestin. Sci. Rep..

[108] Parham K., Sohal M., Petremann M., Romanet C., Broussy A., Van Ba C.T., Dyhrfjeld-Johnsen J. (2019). Noise-induced trauma produces a temporal pattern of change in blood levels of the outer hair cell biomarker prestin. Hear. Res..

[109] Glowatzki E., Cheng N., Hiel H., Yi E., Tanaka K., Ellis-Davies G.C.R., Rothstein J.D., Bergles D.E. (2006). The Glutamate-Aspartate Transporter GLAST Mediates Glutamate Uptake at Inner Hair Cell Afferent Synapses in the Mammalian Cochlea. J. Neurosci..

[110] Sugawara M., Corfas G., Liberman M.C. (2005). Influence of Supporting Cells on Neuronal Degeneration after Hair Cell Loss. J. Assoc. Res. Otolaryngol..

[111] Jang M.W., Lim J., Park M.G., Lee J.H., Lee C.J. (2022). Active role of glia-like supporting cells in the organ of Corti: Membrane proteins and their roles in hearing. Glia.

[112] Graham C.E., Vetter D.E. (2011). The mouse cochlea expresses a local hypothalamic-pituitary-adrenal equivalent signaling system and requires corticotropin-releasing factor receptor 1 to establish normal hair cell innervation and cochlear sensitivity. J. Neurosci..

[113] Tserga E., Damberg P., Canlon B., Cederroth C.R. (2021). Auditory synaptopathy in mice lacking the glutamate transporter GLAST and its impact on brain activity. Prog. Brain Res..

[114] Wan G., Gómez-Casati M.E., Gigliello A.R., Liberman M.C., Corfas G. (2014). Neurotrophin-3 regulates ribbon synapse density in the cochlea and induces synapse regeneration after acoustic trauma. eLife.

[115] May L.A., Kramarenko I.I., Brandon C.S., Voelkel-Johnson C., Roy S., Truong K., Francis S.P., Monzack E.L., Lee F.-S., Cunningham L.L. (2013). Inner ear supporting cells protect hair cells by secreting HSP70. J. Clin. Investig..

[116] Breglio A.M., May L.A., Barzik M., Welsh N.C., Francis S.P., Costain T.Q., Wang L., Anderson D.E., Petralia R.S., Wang Y.-X. (2020). Exosomes mediate sensory hair cell protection in the inner ear. J. Clin. Investig..

[117] Koles L., Szepesy J., Berekmeri E., Zelles T. (2019). Purinergic Signaling and Cochlear Injury-Targeting the Immune System?. Int. J. Mol. Sci..

[118] Basappa J., Graham C.E., Turcan S., Vetter D.E. (2012). The cochlea as an independent neuroendocrine organ: Expression and possible roles of a local hypothalamic–pituitary–adrenal axis-equivalent signaling system. Hear. Res..

[119] Slominski R.M., Tuckey R.C., Manna P.R., Jetten A.M., Postlethwaite A., Raman C., Slominski A.T. (2020). Extra-adrenal glucocorticoid biosynthesis: Implications for autoimmune and inflammatory disorders. Genes Immun..

[120] Xie Y., Tolmeijer S., Oskam J.M., Tonkens T., Meijer A.H., Schaaf M.J.M. (2019). Glucocorticoids inhibit macrophage differentiation towards a pro-inflammatory phenotype upon wounding without affecting their migration. Dis. Model. Mech..

[121] Achuthan A., Aslam A.S.M., Nguyen Q., Lam P.-Y., Fleetwood A.J., Frye A.T., Louis C., Lee M.-C., Smith J.E., Cook A.D. (2018). Glucocorticoids promote apoptosis of proinflammatory monocytes by inhibiting ERK activity. Cell Death Dis..

[122] Wang B., Kasper M., Laffer B., Zu Hörste G.M., Wasmuth S., Busch M., Jalilvand T.V., Thanos S., Heiligenhaus A., Bauer D. (2020). Increased Hydrostatic Pressure Promotes Primary M1 Reaction and Secondary M2 Polarization in Macrophages. Front. Immunol..

[123] Roy M., Sapolsky R.M. (2003). The Exacerbation of Hippocampal Excitotoxicity by Glucocorticoids Is Not Mediated by Apoptosis. Neuroendocrinology.

[124] Sorrells S.F., Munhoz C.D., Manley N.C., Yen S., Sapolsky R.M. (2014). Glucocorticoids Increase Excitotoxic Injury and Inflammation in the Hippocampus of Adult Male Rats. Neuroendocrinology.

[125] Li P., Bing D., Wang S., Chen J., Du Z., Sun Y., Qi F., Zhang Y., Chu H. (2019). Sleep Deprivation Modifies Noise-Induced Cochlear Injury Related to the Stress Hormone and Autophagy in Female Mice. Front. Neurosci..

[126] Shen H., Lin Z., Lei D., Han J., Ohlemiller K.K., Bao J. (2011). Old mice lacking high-affinity nicotine receptors resist acoustic trauma. Hear. Res..

[127] Hickman T.T., Hashimoto K., Liberman L.D., Liberman M.C. (2020). Synaptic migration and reorganization after noise exposure suggests regeneration in a mature mammalian cochlea. Sci. Rep..

[128] Shrestha B.R., Chia C., Wu L., Kujawa S.G., Liberman M.C., Goodrich L.V. (2018). Sensory Neuron Diversity in the Inner Ear Is Shaped by Activity. Cell.

[129] Bustin S.A. (2010). Why the need for qPCR publication guidelines—The case for MIQE. Methods.

[130] Vandesompele J., De Preter K., Pattyn F., Poppe B., Van Roy N., De Paepe A., Speleman F. (2002). Accurate normalization of real-time quantitative RT-PCR data by geometric averaging of multiple internal control genes. Genome Biol..

[131] Suthakar K., Liberman M.C. (2019). A simple algorithm for objective threshold determination of auditory brainstem responses. Hear. Res..

[132] Buran B. (2020). Auditory Wave Analysis, Version 0.0.3.

[133] Jensen J.B., Lysaght A.C., Liberman M.C., Qvortrup K., Stankovic K.M. (2015). Immediate and Delayed Cochlear Neuropathy after Noise Exposure in Pubescent Mice. PLoS ONE.

[134] Fang Q.J., Wu F., Chai R., Sha S.H. (2019). Cochlear Surface Preparation in the Adult Mouse. J. Vis. Exp..

[135] Montgomery S.C., Cox B.C. (2016). Whole Mount Dissection and Immunofluorescence of the Adult Mouse Cochlea. J. Vis. Exp..

[136] Müller M., von Hünerbein K., Hoidis S., Smolders J.W. (2005). A physiological place–frequency map of the cochlea in the CBA/J mouse. Hear. Res..

[137] Schindelin J., Arganda-Carreras I., Frise E., Kaynig V., Longair M., Pietzsch T., Preibisch S., Rueden C., Saalfeld S., Schmid B. (2012). Fiji: An open-source platform for biological-image analysis. Nat. Methods.

[138] Wang Y., Wang C., Ranefall P., Broussard G.J., Wang Y., Shi G., Lyu B., Wu C.-T., Wang Y., Tian L. (2020). SynQuant: An automatic tool to quantify synapses from microscopy images. Bioinform. Oxf. Engl..

[139] Manrique J.F.M., Voit P.R., Windsor K.E., Karla A.R., Rodriguez S.R., Beaudoin G.M.J. (2021). SynapseJ: An Automated, Synapse Identification Macro for ImageJ. Front. Neural Circuits.

[140] Van de Schoot R., Depaoli S. (2014). Bayesian analyses: Where to start and what to report. Eur. Health Psychol..

[141] Wagenmakers E.-J., Lodewyckx T., Kuriyal H., Grasman R. (2010). Bayesian hypothesis testing for psychologists: A tutorial on the Savage–Dickey method. Cogn. Psychol..

[142] Vuorre M. (2017). Sometimes I R: Bayes Factors with brms. https://mvuorre.github.io/posts/2017-03-21-bayes-factors-with-brms/.

[143] Brydges C.R., Gaeta L. (2019). An Introduction to Calculating Bayes Factors in JASP for Speech, Language, and Hearing Research. J. Speech Lang. Hear. Res..

