# Supplementary figures and images for: Conditional Ablation of Glucocorticoid and Mineralocorticoid Receptors from Cochlear Supporting Cells Reveals Their Differential Roles for Hearing Sensitivity and Dynamics of Recovery from Noise-Induced Hearing Loss
Source: Int J Mol Sci. 2023 Feb 7;24(4):3320. doi: 10.3390/ijms24043320 (PMC9961551; doi:10.3390/ijms24043320)

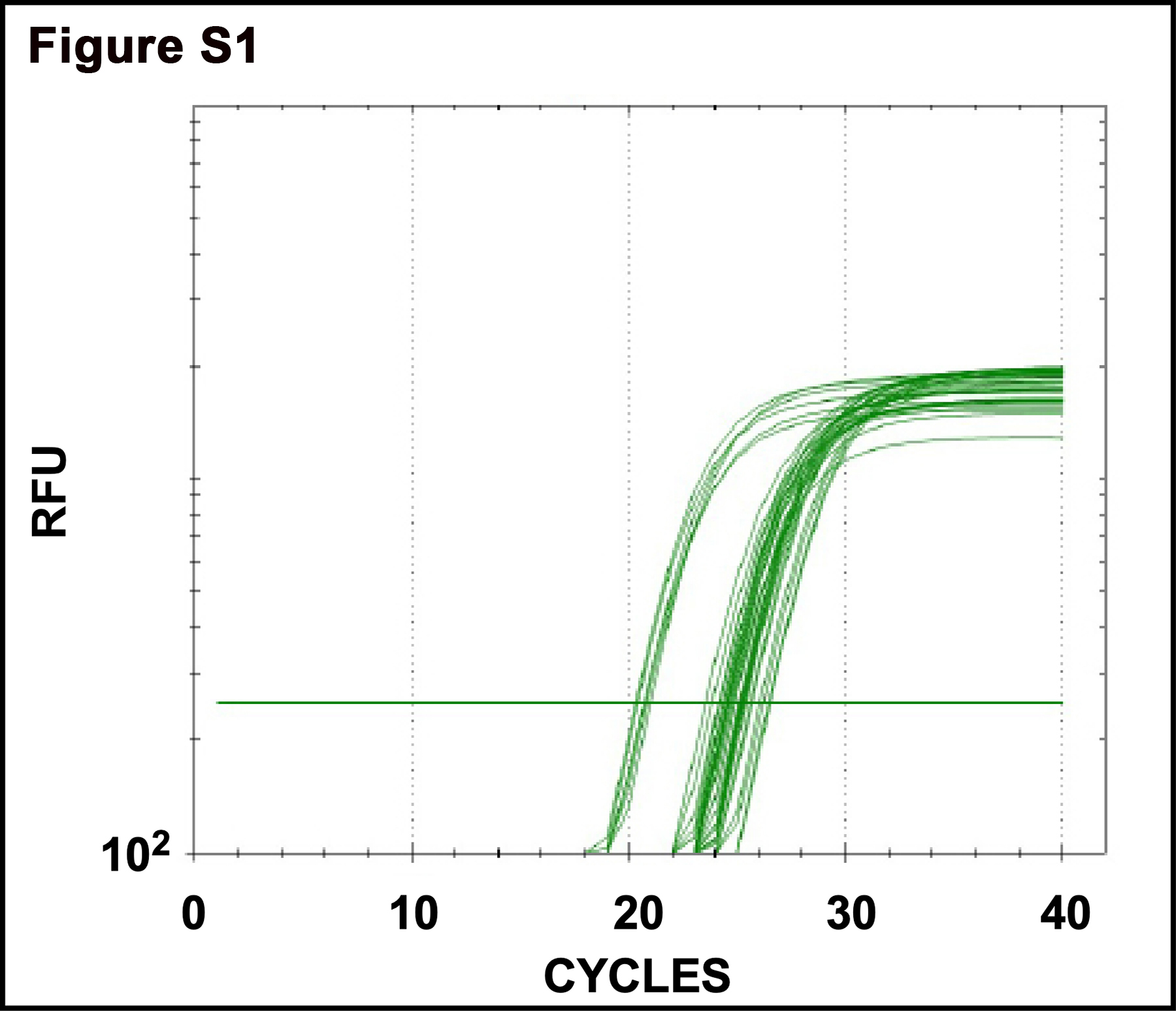

Supplement: Supplementary file 1 [file ijms-24-03320-s001.zip › ijms-2116851-supplementary/Supplementary Figure S1.jpg]

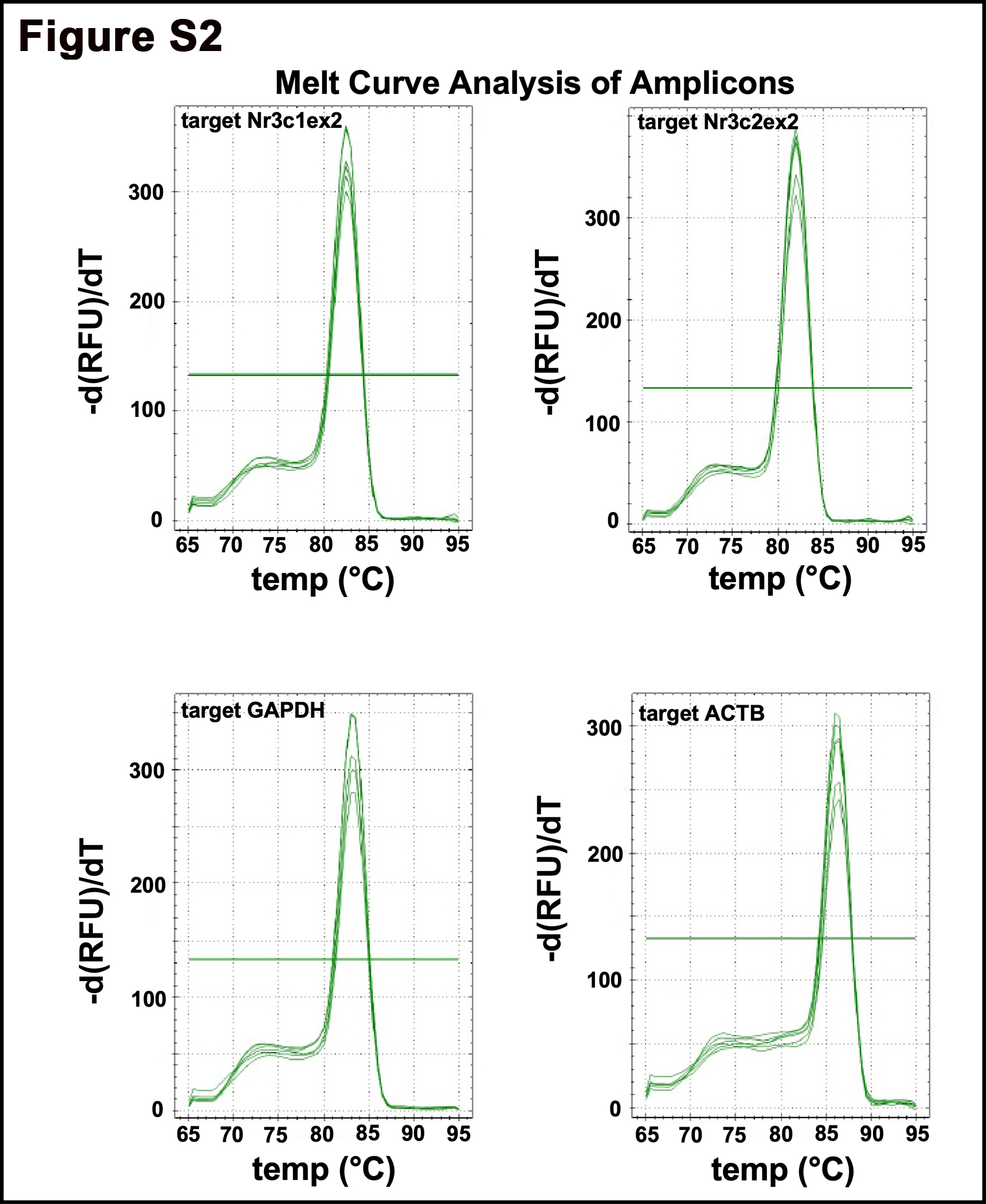

Supplement: Supplementary file 1 [file ijms-24-03320-s001.zip › ijms-2116851-supplementary/Supplementary Figure S2.jpg]

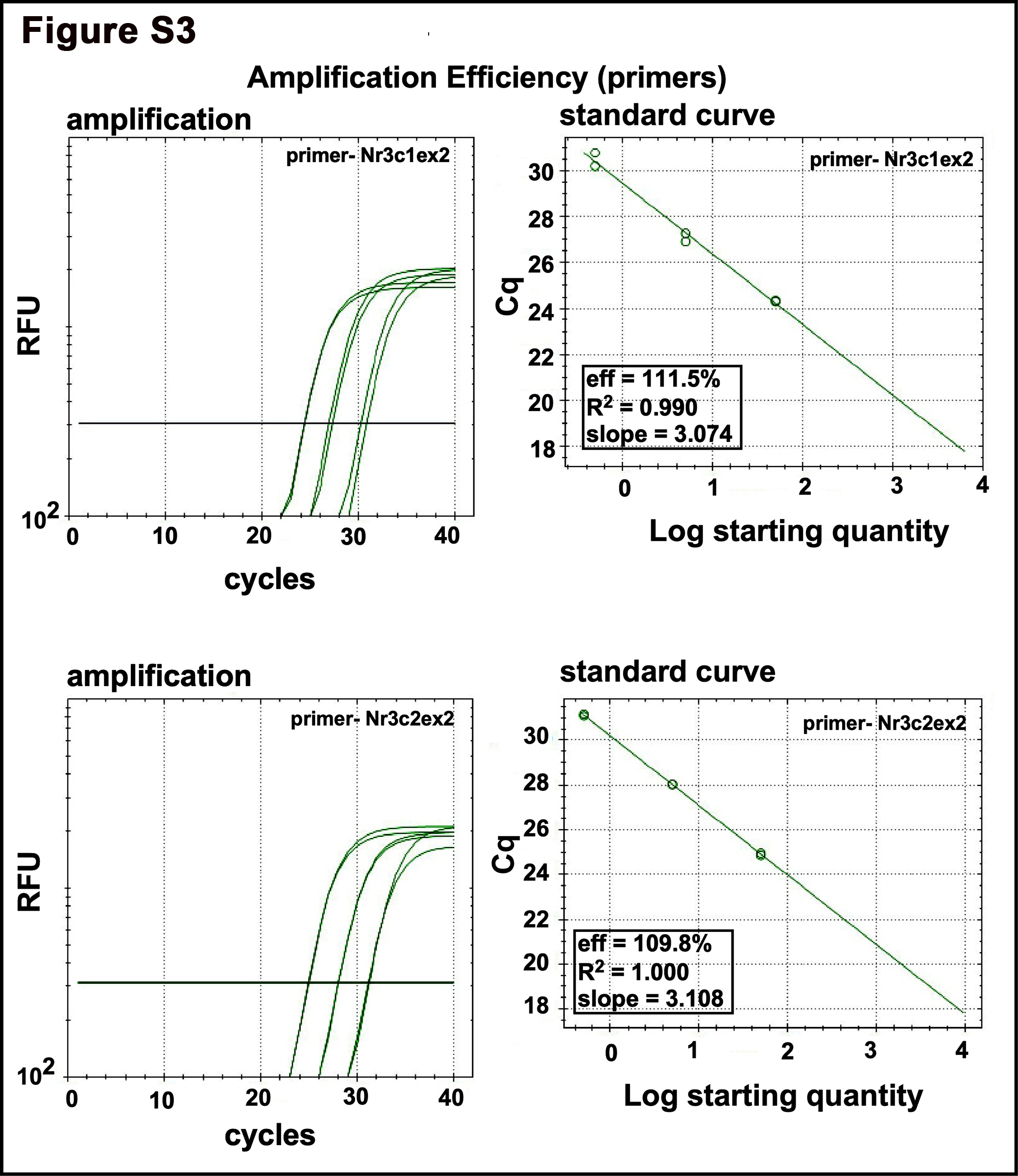

Supplement: Supplementary file 1 [file ijms-24-03320-s001.zip › ijms-2116851-supplementary/Supplementary Figure S3.jpg]

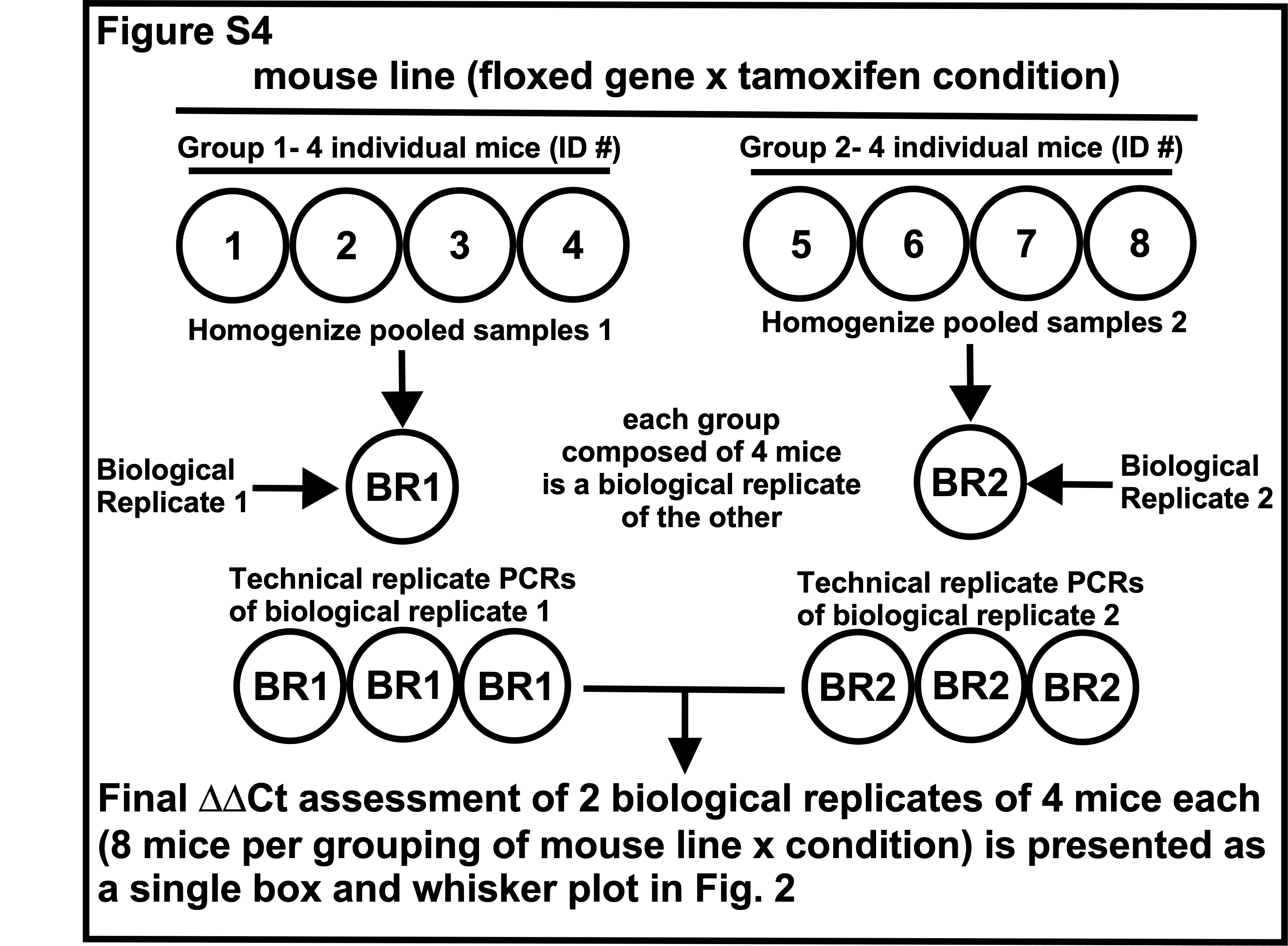

Supplement: Supplementary file 1 [file ijms-24-03320-s001.zip › ijms-2116851-supplementary/Supplementary Figure S4.jpg]

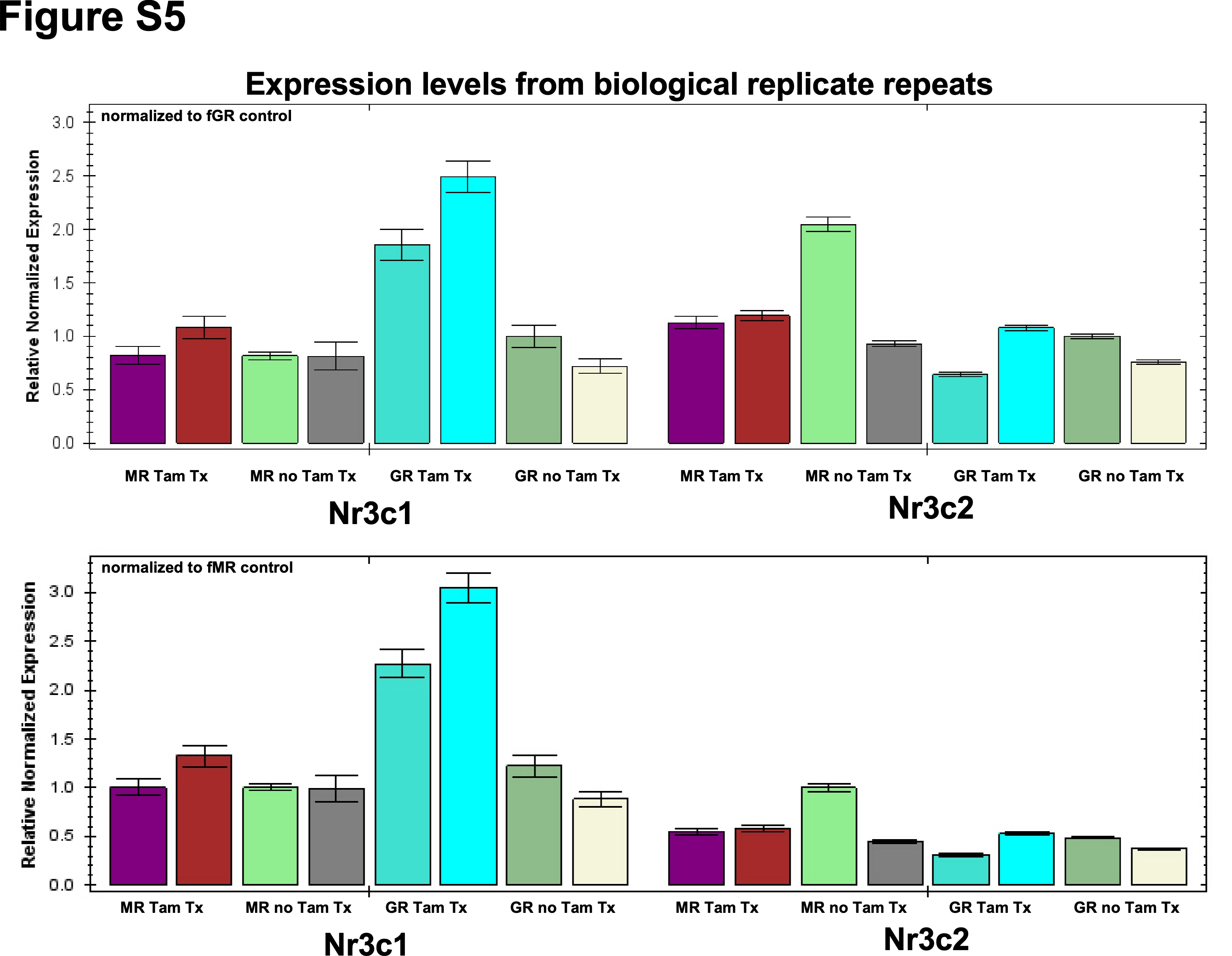

Supplement: Supplementary file 1 [file ijms-24-03320-s001.zip › ijms-2116851-supplementary/Supplementary Figure S5.jpg]
